# The intertwining of world news with Sustainable Development Goals: An effective monitoring tool

**DOI:** 10.1016/j.heliyon.2021.e06174

**Published:** 2021-02-05

**Authors:** Tímea Czvetkó, Gergely Honti, Viktor Sebestyén, János Abonyi

**Affiliations:** a MTA-PE “Lendület” Complex Systems Monitoring Research Group, University of Pannonia, Egyetem str. 10, H-8200 Veszprém, Hungary; b Institute of Advanced Studies Köszeg, Chernel str. 14, H-9730 Köszeg, Hungary; c Sustainability Solutions Research Lab, University of Pannonia, Egyetem str. 10, H-8200 Veszprém, Hungary

**Keywords:** Sustainable development goals, Climate sensitivity, News analysis, Network analysis

## Abstract

This study aims to bring about a novel approach to the analysis of Sustainable Development Goals (SDGs) based solely on the appearance of news. Our purpose is to provide a monitoring tool that enables world news to be detected in an SDG-oriented manner, by considering multilingual as well as wide geographic coverage. The association of the goals with news basis the World Bank Group Topical Taxonomy, from which the selection of search words approximates the 17 development goals. News is extracted from The GDELT Project (Global Database of Events, Language and Tone) which gathers both printed as well as online news from around the world. 60 851 572 relevant news stories were identified in 2019. The intertwining of world news with SDGs as well as connections between countries are interpreted and highlight that even in the most SDG-sensitive countries, only 2.5% of the news can be attributed to the goals. Most of the news about sustainability appears in Africa as well as East and Southeast Asia, moreover typically the most negative tone of news can be observed in Africa. In the case of climate change (SDG 13), the United States plays a key role in both the share of news and the negative tone. Using the tools of network science, it can be verified that SDGs can be characterized on the basis of world news.

This news-centred network analysis of SDGs identifies global partnerships as well as national stages of implementation towards a sustainable socio-environmental ecosystem. In the field of sustainability, it is vital to form the attitudes and environmental awareness of people, which strategic plans cannot address but can be measured well through the news.

## 1 Introduction

News plays a significant role in conveying objectives and major focus areas of both governmental as well as public interests. Furthermore, news can be considered to be creative source of information (Moutidis and Williams, 2019). The level of public awareness and prioritisation regarding sustainability issues can be determined across countries and regions (Barkemeyer et al., 2013). The analysis of news can define areas that gain attention, moreover, governmental strategies and action plans to be identified as sustainable development and environment-related news coverage depend mostly on political institutions and experts as sources of news (Maniou et al., 2017).

News analysis can also play an active role in terms of defining the status of the road map for localizing the SDGs. Generic patterns in sustainability-related media agendas can contribute to support addressing challenges (Barkemeyer et al., 2013). The localization of the SDGs is critical as local spaces are key factors of the successful implementation and preservation of the goals (Taskforce, 2016).

It is our aim to bring about a comprehensive overview of articles concerning sustainable development goals by the different approaches of media analysis. In Section 2. 113 articles are classified according to their relevance with regard to describing the 17 SDGs. The overview of articles revealed a gap in terms of defining SDG areas through news and media appearance and highlighted that there is a need for a systematic tool for country-specific and thematically targeted news-based monitoring of sustainable development.

In this research, the GDELT database (GDELT) is used to explore news about sustainable development goals as well as measure trends and similarities between countries around the world. The proposed methodology can be used to determine which sustainability issues are becoming more critical in a given country or region, or what trends can be observed in the field of sustainability communication.

In connection to world news, the following analytical questions can be formulated:

• What terms directly related to sustainable development goals can be identified in the taxonomic system of news?
• What is the tone of the news regarding each of the sustainability issues?
• What are the differences between countries in terms of SDG news? How similar are the focal points?
• Which countries cooperate in terms of various sustainability issues?

To answer these questions a labelled multilayer network is created to identify the profiles of nations/regions based on the news categorized according to the 17 sustainable development goals and the related targets and indicators.

The novelty of the research is hidden in the broadened exploration of news that overcomes boundaries - it links the multi-level analysis of SDGs with evidence-based events, governmental strategies as well as public interests. The news centred approach can model interconnections between nations regarding sustainable development actions and strategies as well as supports decision-making mechanisms. Furthermore it is possible to measure public awareness, which is closely related to social acceptance and support for the national sustainable development strategies. The appearance of the sustainable development goals in the news has not been directly measurable previously. The main advantage of the developed tool is that the analysis of the occurrence of goals in the published news can be performed in an automated way for any time period.

## 2 Systematic overview of articles describing sustainable development-related topics based on news analysis

This section refers to a systematic overview of articles that used news analysis in order to proximate the sustainable development goals and reveal the extent of their spread, coverage or effect on forming public awareness.

Specific search words were used to identify articles relevant for the overview. In Appendix A, Table A.1 indicates these key search words used to proximate each SDG. The time horizon considered was between 2015 and 2020.

Overall, 112 articles were reviewed, where the selection occurred individually. Table 2, Table 3, Table 4, Table 5, Table 6, Table 7, Table 8, Table 9, Table 10, Table 11 classify articles into the 17 sustainable development goals.

Each table outlines a comprehensive overview of the articles including: a straightforward description of the examined article, methodology used to analyse news, newspapers or search engine used as the basis of the source, the language of news/search, the number of articles examined in the sample, horizontal coverage of data to comprehend its relevance at local, national or international levels, as well as the temporal coverage of data and references.

The exploration of the articles outlined the limitations of SDG-related news analysis, which can be seen in Table 1. In most of the cases, news and media in local/national newspapers/sources disregarding broader geographic coverage, which led up to a narrowing focus with regard to language requirements. Only 28 articles analysed the involvement of at least two countries and 48 explored news in at least two languages (this includes 36 articles where the language of news was not specified).

**Table 1:** Attributes and limitations of the reviewed articles exploring SDG-related news.

Attribute	No. of articles
Total number of reviewed articles	113
Considered at least two countries	29
Considered more than two countries	6
Considered at least two languages^a^	49

a The language was not specified in 36 articles

**Table 2:** Sustainable development goals describing articles based on news analysis - Complex goals.

Complex SDGs							
Description	Method	Analysed news published in	Language	No. of articles	Horizontal coverage of data	Temporal coverage of data	Ref.
**Table:** The article explores how constructive journalism can help move society towards a sustainable future.	Frame analysis	**Table:** UK's Positive News- Environment section/Sustainable Development sub-section	English	54	UK	2016-2017	(Atanasova, 2019)
The article examines how Malaysian online newspapers framed the SDGs.	Thematic analysis, selective coding	**Table:** The Star Online, New Straits Times, Malay Mail Online, Borneo Post Online, Utusan Malaysia, Malaysiakini and Free Malaysia Today	English	16	Malaysia	**Table:** April 2013 - December 2016	(Dauda and Hasan, 2018)
**Table:** The article explore the trends and patterns in sustainability-related media coverage, concentrating on ten main sustainability issues.	**Table:** Three-stage analysis: data collection, automatic modelling, best models based on their trends and seasonal components	**Table:** 23 broadsheet newspapers from Australia, Canada, Germany, UK, US	English and German	230	**Table:** Australia, Canada, Germany, UK and US	2000-2016	(Barkemeyer et al., 2018)
The article explores how media spread information of SDGs to the wider public.	Sequential mixed method	Google News	**Table:** English and Bahasa Indonesia	90	Indonesia	**Table:** August 2016 - August 2018	(Irwansyah, 2018)
**Table:** The article explores how the pattern of media references Millennium Development Goals (MDGs) compares to the emerging early pattern of media references to SustainableDevelopment Goal (SDG).	**Table:** Full text search in LexisNexis, remove duplication, categorization of articles, count of articles per category	LexisNexis search engine -12 newspapers	English	400	**Table:** US, UK, Canada, Hong Kong, Australia, India, Nigeria	**Table:** September 2000 - December 2015	(McArthur and Zhang, 2018)
**Table:** The article examines the divergent and counter intuitive framing of sustainability by British news media.	Qualitative content analysis	**Table:** The Guardian, The Telegraph, The Daily Mail, The Mirror and The Sun (British national newspapers through Lexis Nexis search engine)	English	546	UK	2015	(Diprose et al., 2018)
**Table:** The article explores the role of the media in implementing the SDGs as well as the media coverage of events organised by international development partners in Bangladesh.	Content analysis	15 newspapers - 5 in English, 3 online	English and Bengali	981	Bangladesh	**Table:** July 2015 - December 2018	(Bhattacharya et al., 2020)
**Table:** The article explores the differences between sustainability-related media across countries and regions with greater focus on the relationship of global South and North.	Comparative analysis	LexisNexis - 115 newspapers	8 languages	981	41 countries	**Table:** 2008	(Barkemeyer et al., 2013)

**Table 3:** Sustainable development goals describing articles based on news analysis - SDG 1: No poverty and SDG 2: Zero hunger.

SDG 1: No poverty							
Description	Method	Analysed news published in	Language	No. of articles	Horizontal coverage of data	Temporal coverage of data	Ref.
**Table:** The article explores how the social determinants of health (SDH) have been represented in Canadian news media articles from 1993 to 2014.	Content analysis	ProQuest Database	-	113	Canada	1993 - 2014	(Lucyk, 2016)
**Table:** The article explores local news stories on Portland's tent cities for the homeless.	Content analysis	**Table:** The Oregonian/OregonLive, Portland Tribune, Street Reports	English	189	Portland	2010 - 2016	(Cokeley, 2017)
**Table:** The article examines the coverage of homelessness in the Portuguese press over two periods.	Content analysis	Google Alerts - 69 newspapers	-	287	Portugal	2009 - 2013	(Caeiro and Gonçalves, 2015)
**Table:** The article explores the portrayal of homelessness by the news media in Aotearoa/New Zealand.	Thematic analysis	**Table:** Fairfax Digital, The Dominion Post, The Press, The Sunday Star-Times, Stuff, The New Zealand Herald	-	103	New Zealand	**Table:** June 2013 - June 2015	(Mandeno, 2015)
**Table:** The article explores the media coverage on inequality- related topics on a daily basis and matches it with survey responses that varied daily with respect to the concerns about the economic situation as well as the perceived fairness within the society.	Content analysis	**Table:** Media Tenor International, Tagesschau, Tagesthemen, ZDF heute, ZDF Heute Journal, Bild, Focus, Der Spiegel	-	3867	Germany, Switzerland	**Table:** January 2001 - December 2016	(Diermeier et al., 2017)
**Table:** The article explores media coverage of restrictive immigration legislation and the maintenance of symbolic boundaries.	Iterative frame analysis	**Table:** LexisNexis, ProQuest, or Access World News	-	160	-	2012	(Estrada et al., 2016)
**Table:** The article explores the representation of refugees in British newspapers.	Critical discourse analysis	The Guardian, The Sun	English	-	UK	2015 - 2016	(Kokkonen, 2017)

SDG 2: Zero hunger							
Description	Method	Analysed news published in	Language	No. of articles	Horizontal coverage of data	Temporal coverage of data	Ref.

**Table:** The article explores how the U.S. news media constructs the topic of hunger in Africa for U.S. audiences.	Frame analysis and CDA	**Table:** New York Times, Los Angeles Times, Washington Post and other major newspaper from each state - used Lexis Nexis and NewsBank	English	168	US, Africa	2008-2011	(Kogen, 2015)
**Table:** The article explores the major debate topics and the related issues on the subject of nutrition and food, as well as the main characteristics of the corresponding media coverage.	Thematic content analysis	Google Searce	German	138	Germany	2014-2016	(Rodat et al., 2018)
**Table:** The article explores the public understanding of antioxidants on the Web.	Content analysis	Websites - using Google Search	-	144	-	December 10, 2015	(Aslam et al., 2017)
**Table:** The article explores how debates on school meals and competitive food guidelines debates have been framed at the local and state levels.	Content coding	Nexis database	English	324	US - 11 states	**Table:** August 2012 - August 2015	Nixon et al. (2016)
**Table:** The article examines crisis communication strategies used by four leading Chinese milk companies at various stages of crisis to cope with the largest food safety crisis in China.	Content analysis	Lexis Nexis	-	410	China	**Table:** September 8, 2008 - December 25, 2011	(Zeng et al., 2018)

**Table 4:** Sustainable development goals describing articles based on news analysis - SDG 3: Good health and well-being, and SDG 4: Quality education.

SDG 3: Good health and well-being							
Description	Method	Analysed news published in	Language	No. of articles	Horizontal coverage of data	Temporal coverage of data	Ref.
**Table:** The article explores how the social determinants of health (SDH) have been represented in Canadian news media articles from 1993 to 2014.	Content analysis	ProQuest Database	-	113	Canada	1993 - 2014	(Lucyk, 2016)
**Table:** The article presents an overview of current and emerging sensing and monitoring technologies suitable for precision health, with particular attention given to technologies in high demand such as mobile and portable devices, wearables and implantable sensors.	**Table:** Mixed-methods analysis: systematic review of academic literature, patent databases, news sources	ProQuest and Google News	-	89	-	**Table:** January 2017 - October 2018	(Silvera-Tawil et al., 2019)
**Table:** The article examines historical trends in the reporting of health, illness and medicine in UK and Italian news-papers.	**Table:** Manual and iterative analysis of topics extracted by LDA	The Guardian and La Repubblica	English, Italian	72958	UK, Italy	1984-2017	(Neresini et al., 2019)
**Table:** The article explores the representation of bisexual women who use cannabis.	Inductive qualitative content analysis	Google, Bing, Yahoo, Ask.com, AOL	-	19	-	**Table:** May 2015- October 2015	(Legge et al., 2018)
**Table:** The article analyzes the television reports on World Voice Day.	**Table:** Document analysis technique, statistical analysis	**Table:** World Voice Day (television reports) - through Globo Network	-	45	Brazil	**Table:** March 15 - April 20, 2013	(Dornelas et al., 2015)
**Table:** The article analyses statements in the news made by highly visible political and public figures regarding the subject of rape in the context of themes emerging from ethnography and semi- structured interviews with middle class people in Delhi.	Interview and content analysis	Hindustan Times and Times of India	English	31	India	2011 - 2014	(Edmunds and Gupta, 2016)
**Table:** The article analyses the public discourse on recreational marijuana policy.	Content analysis	42 national and regional, local news outlets	English	610	US	2010 - 2014	(McGinty et al., 2016)
**Table:** The article conducts a topical content analysis of articles on the subject matter of Facebook	**Table:** Content analysis, comparative analysis	Facebook, Communication Source, PsycINFO database	English	475	-	2012 - 2016	(Piotrowski and Fenner, 2017)
**Table:** The article models the epidemiological transmission of the Middle East Respiratory Syndrome (MERS) by news analysis.	**Table:** Granger Causality Test, sensitivity analysis	GDELT, WHO-UN Dataset	-	242+	-	2013-2018	(Balashankar et al., 2019)

SDG 4: Quality education							
Description	Method	Analysed news published in	Language	No. of articles	Horizontal coverage of data	Temporal coverage of data	Ref.

**Table:** The article clusters Twitter data retrieved from the official Twitter accounts of higher education institutions in Indonesia.	**Table:** Cluster analysis, Affinity propagation, hashtag analysis	Twitter	Indonese	31351	Indonesia	2015	(Hamzah and Hidayatullah, 2018)
**Table:** The article explores the bridge between science communication and science education research	Indicative content analysis	New York Times	English	104	US	2010 - 2011	(Shea, 2015)
**Table:** The article discusses content analysis as an important part of the methodological toolbox for elucidating patterns and trends about education policy.	Content analysis	**Table:** New York Times, Wall Street Journal, Washington Post, USA Today	English	-	US	2000 - 2012	(Saraisky, 2016)
**Table:** The article explores the national television news coverage of pre K 12 (early childhood through high school) education in the United States over the past 35 years.	Quantitative content analysis	Vanderbilt Television News Archive	English	2322	US	1980 - 2015	(Coe and Kuttner, 2018)
**Table:** The article explores how newspaper media frames public school closures and what extent does this coverage fulfils the democratic functions of media.	Quantitative data analysis	**Table:** LexisNexis, Access World News, ProQuest, and ProQuest NewStand	English	5452	US	2005 - 2013	(Bierbaum, 2018)
**Table:** The article investigates how the mainstream print media in Kenya covered the teachers, strike, that paralysed learning in primary and secondary schools.	Comparative content analysis	Daily Nation, The Standard	-	58	Kenya	**Table:** December 29, 2014 - January 26, 2015	(Kibet, 2015)

**Table 5:** Sustainable development goals describing articles based on news analysis - SDG 5: Gender equality, and SDG 6: Clean water and sanitation.

SDG 5: Gender equality							
Description	Method	Analysed news published in	Language	No. of articles	Horizontal coverage of data	Temporal coverage of data	Ref.
**Table:** The article explores how the media portrays and represents women during a conflict situation.	Mixed method of content analysis	**Table:** Nagaland Post, The Morung Express, Poknapham, The Sangai Express, The Assam Tribune, Asomia Pratidin	-	183	India	**Table:** Three target events: Manipur (April - July 2010), Nagaland (July - September 2014), Assam (June - July 2004	(Kabi and Nayak, 2019)
**Table:** The article explores how was #metoo covered and framed in Danish and Swedish newspapers and how the similarities and differences between these frames in relation to the political and cultural contexts can be understood.	**Table:** Combination of a quantitative content analysis and qualitative frame analysis	**Table:** Aftonbladet, Dagens Nyheter, Svenska Dagbladet and Expressen in Sweden, and Politiken, Berlingske Tidende, Ekstrabladet and B.T. in Denmark	**Table:** English, Danish, Swedish	879	Denmark, Sweden	15-31 October 2017	(Askanius and Hartley, 2019)
**Table:** The article examines different usages and meanings ascribed to the term gender neutral in news reports over time to elucidate how this multifaceted ideal challenges the gender binary and the omni-relevance of gender faced in everyday life.	Quantitative and qualitative analyses	New York Times, NewsBank	English	959	US	1987 - 2016	(Saguy and Williams, 2019)
**Table:** The article examines the spatial representation of Nigerian female politicians in the news media of Africa's most populous country.	Content analysis	The Guardian, The Punch and Daily Trust	English	109	Nigeria	January - April 2015	(Ette, 2017)
**Table:** The article focuses on the close relationship between social inequalities, orientations of news organizations and news content	Content analysis	**Table:** Le Parisien, Bild, The Sun, Leggo, Blick, NY Daily, France 2, ARD, BBC, RAI, SRF Swiss Info, NPR, TF1, N-TV, Sky GB, Sky IT, CNN, Le Figaro, Die Welt, Daily Mail, Repubblica, Tagesanzeiger, Washington Post, Ouest France, Der Westen, The Times,Corriere della sera, Südostschweiz, LA Times, Le Monde, Frankfurter Allg Zeitung, The Guardian, Il fatto quotidiano, NZZ-, NY Times, Libération, Sueddeutsche, The Telegraph, Unita, Landbote, Tampabay Times, Rue89, Spiegel Online, Open Democracy, Linkiesta, News.ch, Huffington Pos	-	280/country	**Table:** United States, United Kingdom, Germany, Switzerland, France and Italy	June - July 2012	(Humprecht and Esser, 2017)
**Table:** The article explores the situation of “men continue to be overrepresented and woman underrepresented as experts in the media” in Finland.	**Table:** Mixed methods analysis based on three types of data: media, survey, interviews	Helsingin Sanomat, Finnish News Agency, Yleisradio Oy	Finnish	1237	Finland	6 weeks of 2013	(Niemi and Pitkänen, 2017)
**Table:** The article examines and illustrates the ideological struggle of defining ‘feminism’ in mediated discourse analysis.	Textual analysis	**Table:** Huff Post, The New York Times, The Washington Post, Fox News, CNN, MSNBC, Los Angeles Times	English	246	US	20 January 2007 - 31 August 2011	(Loke et al., 2017)

SDG 6: Clean water and sanitation							
Description	Method	Analysed news published in	Language	No. of articles	Horizontal coverage of data	Temporal coverage of data	Ref.

**Table:** The article analyses the coverage of pharmaceutical pollution in the aquatic environment.	Content analysis	**Table:** Chicago Tribune, Denver Post, Los Angeles Times, New York DailyNews, New York Times, USA Today,Wall Street Journal, The Washington Post	English	405	US	2007 - 2014	(Blair et al., 2017)
**Table:** The article explores ageing water infrastructure via a content analysis of newspaper articles over a period of 14 years	Media discourse and content analysis	LexisNexis database	-	500	US	1999 - 2012	(Vedachalam et al., 2016)
**Table:** The article analyses the role played by the print media in creating awareness among the Indian public regarding the paramount sanitation issues.	Content analysis	The Hindu and The Times of India	English	60	India	October 1 - October 31 2014	(Showkat, 2016)
**Table:** The article examines the newspaper coverage of sanitation in Kannada.	Content analysis	Gulbarga: Prajavani and VijayaKarnataka	-	45	Kannada	October 2014 - March 2015	(Kakade, 2015)

**Table 6:** Sustainable development goals describing articles based on news analysis - SDG 7: Affordable and clean energy, and SDG 8: Decent work and economic growth.

SDG 7: Affordable and clean energy							
Description	Method	Analysed news published in	Language	No. of articles	Horizontal coverage of data	Temporal coverage of data	Ref.
**Table:** The article explore how does media portrayal of smart grid (SG) compare in the U.S. and Canada.	Comparative media analysis	**Table:** The Wall Street Journal, USA Today, and The New York Times, Globe and Mail, the National Post and La Presse	English, French	590	Canada, US	1998 - 2013	(Mallett et al., 2018)
**Table:** The article explores long-term newspaper coverage of biogas.	**Table:** Quantitative content analysis, discourse analytic	Helsingin Sanomat, Maaseudun Tulevaisuus	Finnish	435	Finland	2000-2017	(Lyytimäki, 2018)
**Table:** The article explores the features of provincial solar energy development, and their concerns about solar energy.	Network analysis	LocoySpider	Chinese	16893	China	2010 -2014	(Guan et al., 2017)
**Table:** The article examine representations of natural gas development through a content analysis of six regional newspapers in the northern United States.	Content analysis	Youngstown Vindicator, Canton Repository	English	329	US	**Table:** January 1, 2009 - December 31, 2014	(Ashmoore et al., 2016)
**Table:** The article explores the Indigenous Peoples and their involvement in renewable energy.	Content and critical discourse analyses	**Table:** Canadian Major Dailies and Google News, CBC, Globe and Mail,National Post,Vancouver Sun, Toronto Star, APTN	English	153	Canada, Indigeneus	**Table:** November 2008 - November 2017	(Walker et al., 2019)
**Table:** The article shows whether or not an assumed analogue of climate change –energy storage –is as politicized in the traditional media	**Table:** Automated and handcoded content analysis	New York Times and The Guardian	English	900	US, UK	-	(Shapiro, 2018)
**Table:** The article examines the nexus of three trends in electricity systems transformations underway worldwide the scale-up of renewable energy, regionalization, and liberalization.	**Table:** Mixed method analysis including news analysis	Google search, Google news, LexisNexis	-	396	Mediterrian region	2013	(Moore, 2015)

SDG 8: Decent work and economic growth							
Description	Method	Analysed news published in	Language	No. of articles	Horizontal coverage of data	Temporal coverage of data	Ref.

**Table:** The article analyses how online public diplomacy in two Western Balkans states, Croatia and Serbia, framed the issue of unemployment through official government websites.	Content analysis	**Table:** Online news releases in English published on Croatian and Serbian Government websites	**Table:** English, Croatian and Serbian	339	Croatia, Serbia	**Table:** 1 January 1, 2009 - December 31, 2014	(Lusa and Jakopovic, 2017)
**Table:** The article investigates whether news coverage about unemployment affects people s perceptions of the state of the economy.	Quantitative analysis	**Table:** DIGAS, Nexis, and Genios, Bild, Frankfurter Allgemeine Zeitung, Frankfurter Rundschau, Handelsblatt, Süddeutsche Zeitung, TAZ, and Welt and regional new papers	-	7359	Germany	2005 - 2014	(Garz, 2018)
**Table:** The article examine the association between macro- economic news and stock market returns.	Theory of copulas, estimation	**Table:** Thomson Reuters Newswires (TRN) and the Dow Jones Energy Service (DJES)	-	19739	US	**Table:** January 1999 - April 2014	(Medovikov, 2016)
**Table:** The article analyse whether US news on inflation and unemployment causes returns and volatility of seven emerging Asian stock markets.	**Table:** Detection on nonlinear causality via a hybrid approach	**Table:** Bureau of Economic Analysis (BEA), Bureau of Labor Statistics (BLS)	-	229	**Table:** India, Indonesia, South Korea, Philippines, Singapore, Taiwan, Thailand, US	**Table:** November 1, 1994 - June 24, 2014	(Balcilar et al., 2017)

**Table 7:** Sustainable development goals describing articles based on news analysis - SDG 9: Industry, innovation and infrastructure, and SDG 10: Reduced inequalities.

SDG 9: Industry, innovation and infrastructure							
Description	Method	Analysed news published in	Language	No. of articles	Horizontal coverage of data	Temporal coverage of data	Ref.
**Table:** The article argues that local news requires a different method and infrastructure support for effective georeferencing.	Content analysis	Centre Daily Times	-	600	-	**Table:** January 1997 - February 1997	(Cai and Tian, 2016)
**Table:** The article explores, proposes and tests a system analytics framework based on social sensing and text mining to detect topic evolution associated with the performance of infrastructure systems in disasters.	Social sensing and text mining	Twitter	-	63263	**Table:** 150-mile radius of Houston	**Table:** August 2017 - September 2017	(Fan et al., 2018)
**Table:** The article examines the extent to which entrepreneurial innovation is covered in Nigerian national newspapers and how this can lead to sustainable development in Nigeria.	Content analysis	**Table:** Vanguard, The Punch, The Guardian, Business Day	-	1122	Nigeria	2013-2015	(Amodu et al., 2016)
**Table:** The article analyzes the impact of media coverage on opinion leading newspapers and television channels in Germany on new cars registrations	Panel data technique	Media Tenor International	-	6887	Germany	**Table:** March 2001 - October 2011	(Dewenter et al., 2016)
**Table:** The article explores newspapers, representations of different actors in infrastructure projects, and analyses the power relations between them through a case study in Hong Kong.	Critical discourse analysis	South China Morning Post, Apple Daily	**Table:** English, Chinese	500	China	**Table:** October 1, 2008 - February 28, 2010	(Lee and Silva, 2017)
**Table:** The article reviews the possibilities of text mining in the area of cybercrime in digital healthcare.	Text mining, statistical methods	GDELT	**Table:** German, English	300000	-	**Table:** January 2015 - March 2019	(Bertl, 2019)

SDG 10: Reduced inequalities							
Description	Method	Analysed news published in	Language	No. of articles	Horizontal coverage of data	Temporal coverage of data	Ref.

**Table:** The article examines the portrayal of refugees in the United States by comparing four online news outlets.	**Table:** Quantitative content analysis, five frequent frames	Fox News, Breitbart, CNN, The New Yorker	English	-	US	2016	(Issac, 2017)
**Table:** The article explores the link between mass-media coverage of migration and immigration concerns.	Empirical	Media Tenor International	-	3369	**Table:** Germany, Switzerland	**Table:** January 2009 - December 2014	(Benesch et al., 2019)
**Table:** The article explores media coverage of inequality-related topicson a daily basis and matches them with daily varying survey responses with respect to concerns about the economic situation as well as the perceived fairness within the society.	Content analysis	**Table:** Media Tenor International, Tagesschau, Tagesthemen, ZDF heute, ZDF heute Journal, Bild, Focus, Der Spiegel	-	3867	**Table:** Germany, Switzerland	**Table:** January 2001 - December 2016	(Diermeier et al., 2017)
**Table:** The article explores media coverage of restrictive immigration legislation and the maintenance of symbolic boundaries.	Iterative frame analysis	LexisNexis, ProQuest, Access World News	-	160	-	2012	(Estrada et al., 2016)
**Table:** The article explores the textual and visual representations of climate change-induced migration within online news media in the UK.	**Table:** Critical discourse analysis (CDA)	**Table:** Media Watch on Climate Change Tool (http://www.ecoresearch.net/climate/) - The Sun, Daily Mail (Daily Mail Online), The Daily Mirror (Mirror.co.uk), the Telegraph, The Guardian, and the Independent	English	45	UK	-	(Sakellari, 2019)
The article explores gender bias in the news media.	Textual and content analyses	RSS feed	English	885573	-	**Table:** October 19, 2014 - January 19, 2015	(Jia et al., 2015)

**Table 8:** Sustainable development goals describing articles based on news analysis - SDG 11: Sustainable cities and communities, and SDG 12: Responsible consumption and production.

SDG 11: Sustainable cities and communities							
Description	Method	Analysed news published in	Language	No. of articles	Horizontal coverage of data	Temporal coverage of data	Ref.
**Table:** The article explores trends of topics and issues about smart factories within the online news articles.	Text mining-based analysis, ARM, LDA	Naver News	Korean, English	84	Korea	2014-2017	(Jung and Chang, 2018)
**Table:** The article explores the media coverage of air pollution risks and current policies in India.	Content analysis	Google News and Meltwater	English	500	India	**Table:** January 1, 2014 - October 31, 2015	(Murukutla et al., 2017)
**Table:** The article explores news about smart cities on Kompas Online, Indonesia.	Quantitative descriptive research	Kompas Online	English	38	Indonesia	2015	(Yuliarti et al., 2016)
**Table:** The article explores notion of city-making by explicating its communicative processes and functions within the press.	**Table:** Quantitative content analysis; quantitative textual analysis	Miami Herald, Mialmi-Dade Country	English	51	Miami	**Table:** January 1, 2011- January 1, 2014	(Shumow and Gutsche, 2016)

SDG 12: Responsible consumption and production							
Description	Method	Analysed news published in	Language	No. of articles	Horizontal coverage of data	Temporal coverage of data	Ref.

**Table:** The article explores the roles of corporations and a monitoring group in building the corporate social responsibility (CSR) agenda in the news media.	Content coding	LexisNexis, The Wall Street Journal, The New York Times	English	12603	US	**Table:** January 1, 2008 - December 31, 2010	(Lee and Riffe, 2017)
**Table:** The article explores long-term newspaper coverage of biogas.	**Table:** Quantitative content analysis, discourse analytics	Helsingin Sanomat, Maaseudun Tulevaisuus	Finnish	435	Finland	2000-2017	(Lyytimäki, 2018)
**Table:** The article analyzes how electronic waste (e-waste) gets represented in television news stories.	**Table:** Social semiotics and multimodal discourse analysis	CNN, BBC, BBC1, CCTV Africa Live, CBS	English	-	-	**Table:** May 30, 2013- February 15, 2014	(Andersson, 2017)
**Table:** The article explores the prominence of the corporate responsibility of the media with regards to firms.	Text analytics, content analysis	GDELT database	-	554	Singapore	**Table:** May 2015 - May 2016	(Azhar et al., 2019)
**Table:** The article examines the nexus of three trends in transformation of electricity systems underway worldwide, e.g. the scale-up of renewable energy, regionalization, and liberalization.	**Table:** Mixed method analysis including news analysis	Google Search, Google News, LexisNexis	-	396	Mediterranean region	2013	(Moore, 2015)

**Table 9:** Sustainable development goals describing articles based on news analysis - SDG 13: Climate action.

SDG 13: Climate action							
Description	Method	Analysed news published in	Language	No. of articles	Horizontal coverage of data	Temporal coverage of data	Ref.
**Table:** The article explores the textual and visual representations of climate change-induced migration within online news media in the UK.	Critical discourse analysis (CDA)	**Table:** Media Watch Change Tool (http://www.ecoresearch.net/climate/) - Sun, The Daily Mail (Daily Mail Online), The Daily Mirror (Mirror.co.uk), the Telegraph, The Guardian, The Independent	English	45	UK	-	(Sakellari, 2019)
**Table:** The article describes the climate change-related media coverage in India over 20 years.	Automated content analysis	The Times of India and The Hindu	English	18224	India	**Table:** January 1, 1997- December 31, 2016	(Keller et al., 2020)
**Table:** The article explores how carbon capture and storage as well as biomass can be beneficial with regard to the mitigation of climate change by news media.	SPEED Framework	**Table:** LexisNexis - The Boston Globe, The Star Tribune, The Billings Gazette, Houston Chronicle, The Republican, St. Paul Pioneer Press, Missoulian, Austin American- Statesman, Cape Cod Times, Duluth News Tribune, Bozeman Daily Chronicle, and Midland Reporter-Telegram	English	216	**Table:** US (Massachusetts, Minnesota, Montana and Texas)	**Table:** January 1, 1990 - June 15, 2009	(Feldpausch-Parker et al., 2015)
**Table:** The article examines climate-change news coverage between 1997 and 2010 in Canada.	**Table:** Analyse longitudinal trends in articles, analyse peak periods	The Globe and Mai, National Post	English	**Table:** 8960; 603	Canada	**Table:** 1997-2010; 2007-2008	(Stoddart et al., 2016)
**Table:** The article explores the comparision between social media and mainstream news on climate change	**Table:** Mixture of annotation conducted by the authors and crowdsourced workers through the CrowdFlower platform	GDELT, Twitter,	limited to English	**Table:** 561644, 482615	World	**Table:** September 1, 2013 - September 31, 2014	(Olteanu et al., 2015)
**Table:** The article explores the importance of the role played by media analysis in how political representations in international negotiations will develop.	Frame analysis, comparative analysis	**Table:** Business Day, The Mercury; Dagens Naeringsliv, Bergens Tidende	English	266; 62	**Table:** South Africa, Norway	**Table:** During the Seventeenth Conference of the Parties (COP17) -2011	(Johannessen, 2015)
**Table:** The article explores climate change communication both as a news product and cultural phenomenon.	Frame analysis	Prime News, TV3 News, One News	English	592	New Zealand	**Table:** August 2, 2012 - August 22, 2012	(Bourk et al., 2017)
**Table:** The article explores the dissonance between global and a specific local environmental imaginary through a case study of community newspaper coverage.	Content analysis	The Advertiser	English	20	**Table:** Boksburg, Gauteng, South Africa.	**Table:** January 2014 - July 2015	(Lawhon et al., 2018)
**Table:** The article explores the structure of the public discourse concerning the Gateway project.	Open coding, frame analysis	**Table:** LexisNexis and Canadian Newsstand database - Postmedia Network, Toronto Star, Globe and Mail, Glacier Media	-	853	Canada	**Table:** December 2011; January 2012; May-June 2012	(Raso and Neubauer, 2016)
**Table:** The article shows whether or not an assumed analogue of climate change energy storage is as politicized in the traditional media	**Table:** Automated and handcoded content analysis	The New York Times, The Guardian	English	900	US, UK	-	(Shapiro, 2018)
**Table:** The article explores the media's role in bridging the information gap concerning environmentally sustainable development.	Critical discourse analysis	**Table:** Sunday Mail, The Patriot, The Herald, Newsdaym Financial Gazette	-	30	Zimbabwe	**Table:** January 2012 - April 2016	(Zhou et al., 2017)
**Table:** The article analyses digital and broadcast news media coverage of the Fourth National Climate Assessment in order to get a sense of ethos constructions in climate -change communication.	Comparative rhetorical analysis	**Table:** The Washington Post, USA Today, CBS News, Fox News, CNN, The New York Times, BuzzFeed News, Los Angeles Times, Reuters (via Yahoo News), The Guardian (US Edition), Huff Post, San Francisco Chronicle, National Geographic	English	14	US	2018	(Dakota Rohlin et al., 2019)

**Table 10:** Sustainable development goals describing articles based on news analysis - SDG 14: Life below water and SDG 15: Life on land.

SDG 14: Life below water							
Description	Method	Analysed news published in	Language	No. of articles	Horizontal coverage of data	Temporal coverage of data	Ref.

**Table:** The article explores the role of mass media in the diffusion of marine conservation information.	Content analysis	**Table:** Google Search, La Tercera, Canal 13, MEGA, CHV, TVN	Spanish	-	Chile	2011-2013	(Thompson-Saud et al., 2018)
**Table:** The article examines the coverage of aquaculture in regional and national newspapers	Content coding	**Table:** The Advocate (Louisiana), The Portland Press Herald (Maine), The Boston Globe (Massachusetts) The New York Times, The Washington Post, The Wall Street Journal, USA Today	English	493	US	2005-2015	(Rickard and Feldpausch-Parker, 2016)
**Table:** The article explores the presence of marine issues in the news.	Content analysis	Público	Portuguese	1309	Portugal	**Table:** October 2002 - December 2010	(Pinto et al., 2020)
**Table:** The article examines the construction of the China-Pakistan Economic Corridor issue in Indian media through the discourse analysis of news.	Discourse analysis	**Table:** Telegraph India, Deccan Herald, The Tribune, Hindustan Times, The Hindu, The Times of India	-	44	Pakistan, India	**Table:** April 20, 2015 - June 30, 2015	(Khan et al., 2016)

SDG 15: Life on land							
Description	Method	Analysed news published in	Language	No. of articles	Horizontal coverage of data	Temporal coverage of data	Ref.

**Table:** The article explores the media coverage of online news to analyze existing media representation of forest management	Qualitative content analysis	Google Alerts	German	613	-	**Table:** January 13, 2016 - January 12, 2017	(Ranacher et al., 2019)
**Table:** The article analyzes how the news media influences the construction of the social perception of forests and forestry.	**Table:** Summative content analysis - combination of both quantitative and qualitative data analysis	El País, El Mundo	Spanish	1870	Spain	2009-2012	(Fabra Crespo and Rojas Briales, 2015)
**Table:** The article explores newspapers, framing of urban forests, focusing on if and how the framing changed as a result of a major storm that highlighted urban forest disservices.	Content analysis	**Table:** Toronto Star, Mississauga News, Brampton Guardian	English, French	595	Ontario, Canada	**Table:** January 1, 2013 - December 31, 2014	(Conway and Jalali, 2017)
**Table:** The article explores oil and gas drilling proposals in the Arctic National Wildlife Refuge (ANWR).	Content analysis	**Table:** LexisNexis: Wall Street Journal, The New York Times, USA Today, Alaskan local newspapers	English	100	Alaska, US	1984-2014	(Kroner, 2016)
**Table:** The article explores wildlife-related news coverage by the Indian print news agencies and quantifies its patterns.	**Table:** Content analysis - General linear models	49 different newspapers	English	766	-	2011	(Lyngdoh et al., 2017)
**Table:** The article explores how the conflict between wildlife and humans was framed by the news media.	**Table:** Designed Market Area sampling method	**Table:** Selected by DMA sampling method - 3 newspapers/ DMA	-	392	US	2010-2015	(Stafford et al., 2018)
**Table:** The article explores the environmental coverage by the Nigerian press and examines the factors that affect coverage.	**Table:** Sequential mixed methods for content analysis and in-depth interviews	**Table:** The Guardian, Business Day, Daily Trust, ThisvDay	-	754	Nigeria	**Table:** January 2013 - December 2014	(Ogadimma et al., 2018)

**Table 11:** Sustainable development goals describing articles based on news analysis - SDG 16: Peace, justice and strong institutions and SDG 17: Partnerships for the goals.

SDG 16: Peace, justice and strong institutions							
Description	Method	Analysed news published in	Language	No. of articles	Horizontal coverage of data	Temporal coverage of data	Ref.
The article examines conflict intensity in Arab countries.	Remote sensing	GDELT, Flicker	-	-	Arab countries	**Table:** Arab Spring: January 2007, November 2010, December 2010-2018	(Levin et al., 2018)
**Table:** The article introduces a protest news framing cycle and presents the results of a longitudinal analysis of news attention and framing of protest movements.	Content analysis, discourse analysis	LexisNexis, The New York Times	English	228	-	September 2011 - July 2014	(Gottlieb, 2015)
**Table:** The article examines the framing of visual images of conflicts and violence in television-news programming.	Content analysis	**Table:** Al Jazeera, Al Jazeera English, Al Arabiya, Alhurra, BBC Arabic	Arabic, English	6595	Arab countries	August 1, 2010 - June 15, 2011	(Bruce and Conlin, 2016)
**Table:** The article explores how the media portrays and represents conflicts and the restoration of peace as well as women in conflict situations.	Content analysis	**Table:** Nagaland Post, The Morung Express, Poknapham, The Sangai Express, The Assam Tribune, Asomiya Pratidin	Hindi, English	183	India	-	(Kabi and Nayak, 2019)
**Table:** The article examines the influence on of IBSA Dialogue Forum (India, Brazil, South Africa) membership South Africa.	Qualitative framing analysis	NewsBank database	-	110	South Africa	June 6, 2003 - September 30, 2016	(Rosas-Moreno, 2018)
**Table:** The article explores media coverage of major conflicts as well as the war/peace frame of news production/ presentation.	Content analysis	**Table:** The Globe and Mail, National Post, Toronto Star, Toronto Sun, Jerusalem Post, The New York Times, New York Post	English	522	**Table:** Afghanistan and Israeli-Hezbollah wars	2016 July - September	(Hackett and Schroeder, 2017)
**Table:** The article examines how the same event the Syrian conflict - has been covered by US and Chinese media.	Critical discourse analysis	The New York Times, China Daily	-	397	Syria, US, China	March 2011 - February 2012	(Thanaphokhai, 2015)

SDG 17: Partnership for the goals							
Description	Method	Analysed news published in	Language	No. of articles	Horizontal coverage of data	Temporal coverage of data	Ref.

**Table:** The article presents findings from a media analysis of mainstream newspaper coverage of the Trans-Pacific Partnership Agreement (TPP)	Content analysis	**Table:** Factiva- algaryHerald, The Edmonton Journal, The Globe and Mail, National Post, Ottawa Citizen, The Province, The Star Phoenix, Times Colonist, Toronto Star, Vancouver Sun, Windsor Star, and Winnipeg Free Press.	English	404	Canada	January 2010 - June 2014	(Schram et al., 2016)
**Table:** The article explores how German and Russian media represents the relationship between EU - Ukraine.	Content analysis	**Table:** Süddeutsche Zeitung, Frankfurter Allgemeine Zeitung, Kommersant and Rossiyskaya Gazeta	German, Russian	160	**Table:** Germany, Russia, Ukraine, EU	**Table:** 4 periods: April 30 - May 10, 2009; September 22 - October 3, 2011; November 21 - December 2, 2013, May 14-25, 2015	(Kleinschnitger et al., 2018)
**Table:** The article explores the territorial and temporal patterns of the media coverage of EU cohesion policy.	**Table:** Natural language processing techniques, Sentiment analysis	**Table:** The Telegraph, The Guardian, El País, El Mundo, The Scotsman La Voz de Galicia, Financial Times, Politico, EURACTIV	English, Spanish	4000	**Table:** EU - focus on Spain and UK	2010-2017	(Mendez et al., 2020)
**Table:** The article analyses the international economic news about Chinese outward foreign direct investment in Latin American countries from corresponding Latin American newspapers	Content analysis	**Table:** Factiva database: La Nación, La Voz, O Globo, Folha de S. Paulo, El Mercurio, La Tercera, Portafolio, El Espectador, El Universal, La fomada, El Comercio, El Nacional, El Universal, La Estrella	Spanish	602	China, Latin America	January 1, 2008 - December 31, 2014	(Zhu and Wang, 2018)
**Table:** The article explores the role of the media in implementing the SDGs as well as the media coverage of events organised by international development partners in Bangladesh.	Content analysis	15 newspapers - 5 in English, 3 online	English and Bengali	981	Bangladesh	July 2015 - December 2018	(Bhattacharya et al., 2020)

Furthermore, the time period of the analysis considered was typically far earlier than the day of publication, which on the one hand, is an essential way to gain overall knowledge of a closed period, on the other hand to advance news analysis to the next level to serve as an effective monitoring tool, temporal coverage must be as recent as possible. This enables present issues to be detected and assumptions as well as actions taken to meet the sustainable development goals.

This finding led to the provision of a tool that enables sustainable development-specific world news to be monitored in several languages and considered the latest data available, for which the GDELT Project served as an effective source.

## 3 Development of the methodology of the news-related analysis of sustainable development goals

In this section, the methodological steps are discussed from data acquisition and categorization up until the formation of multi-layered networks. The proposed methodological workflow is suitable to build multi-layered networks to reveal the focal points between news and specific contents and taxonomies. The key assumption is that connecting topic-specific news and their spatial connection can reveal the sensitivity of country to a given topic as well as detect interconnections between keywords. Regarding the analysis sustainable development goals the spatial coverage is considered at the country level, while the temporal horizon is considered in the year of 2019. This chapter presents the connection between the specific content of sustainable development goals and the taxonomy of the World Bank as well as the segments of the My World 2015 survey, followed by a presentation of the development of analytical Structured Query Language (SQL) queries. The reader is then guided through the description of the network formulation from the extracted information to determine the news and SDG.

### 3.1 The workflow of SDG-related acquisition of news gained from the GDELT Project

**Figure 1:**
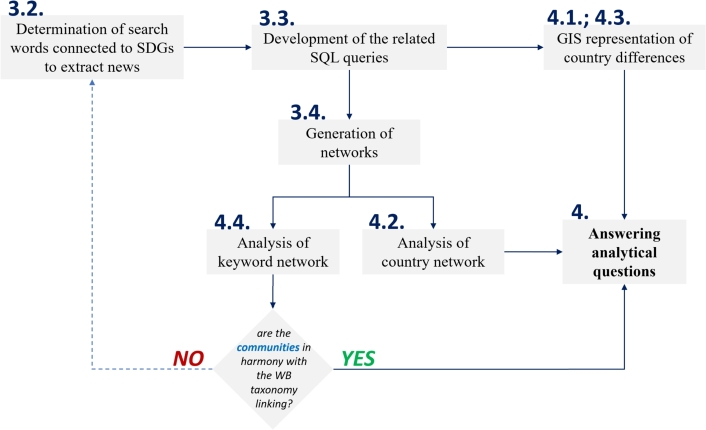
The proposed workflow of the news-based analysis of sustainability issues.

The steps of the analysis are summarized in Fig. 1, where the dark blue numbers refer to the related subsections in the paper.

World news on sustainable development goals is a good illustration of the mentality of countries' concerning the 2030 Agenda. The published news stories are labelled along with the ontologies in the GDELT Project, which allows for their thematic analysis if the ontologies related to the given issue are selected, in our case, the SDGs based on the taxonomy of the World Bank.

The taxonomy of the World Bank has been reviewed and the categories related to SDGs selected.

The data were extracted from the GDELT's Global Knowledge Graph (GKG). GDELT has already been used to estimate the future level of violence in Afghanistan (Yonamine, 2013), explore disasters and their determinants (Kwak and An, 2014), determine the risk of upcoming social unrest events and predict indicators related to the instabilities of country (Qiao and Chen, 2016) or to analyze peace and conflict data (Keertipati et al., 2014).

Two methods were used for the extraction, namely the “standard” way, which involves Google Big-Query engine, and a self-developed Python-based engine. For short queries, and validation, Big-Query was used, for more analytical and long time frame analysis, the offline Python-based engine was used.

The results of the queries can also be analyzed using Geographic Information System (GIS) techniques, thus revealing how the countries are sensitive to SDG issues and how much news is reported about SDGs. Multidimensional networks comprised of queries aim to shed light on the relationships between countries and topics. The ontological assignments with regard to the SDGs can be checked based on the identified keyword communities, however, it is important to emphasize that due to the interconnectedness of SDGs the validation requires expert knowledge.

### 3.2 Determination of search words connected to SDGs to extract news

WBG Topical Taxonomy refers to the World Bank Group's topical knowledge domains and areas of expertise, whilst the segments determined by the My World 2015 survey contributed to the formation of Agenda 2030. These data was used as a basis to approximate these categories within the 17 goals - involved targets and its indicators.

The selection from topics with regard to WBG Topical Taxonomy and association with the development goals happened through expert sampling. These associated search words are shown in Appendix B.

The segments of the My World 2015 survey are the following: A good education, Better healthcare, Better job opportunities, An honest and responsive government, Affordable and nutritious food, Protection against crime and violence, Access to clean water and sanitation, Support for people who can't work, Better transport and roads, Equality between men and women, Reliable energy at home, Political freedoms, Freedom from discrimination and persecution, Protecting forests, rivers and oceans, Phone and Internet access, and Action taken on climate change.

The formulation of sustainable development goals is concise and ambitious, therefore, the World Bank categories are preferably linked on the basis of the keywords in the more detailed description of the indicators. For example, the following keywords can be extracted from the indicators of SDG 15: ‘ecosystem’, ‘forest’, ‘land’, ‘protected’, ‘biodiversity’, ‘red list’, ‘mountain’, ‘wildlife’ and ‘species’. Links are based on expert knowledge, however, the methodology allows them to be validated.

For SDG 13, “Take urgent action to combat climate change and its impacts”, 5 targets and 7 indicators were declared. To measure this goal, the number of countries with disaster risk reduction strategies, number of affected persons by disaster, mobilized financial support and other policy strategic measures are used. Table 12 shows that topics were selected that are very closely related to the concept of SDG 13 and its indicators. The following keywords extracted from the SDG 13 targets and indicators that linked with the World Bank categories: ‘disaster’, ‘natural disaster’, ‘policies, strategies and planning’, ‘education’, ‘climate change mitigation and adaptation’, ‘impact reduction’, ‘early warning’. The experience of studies discussing the interconnectedness of SDGs was taken into consideration in the application of the search words (Sebestyén et al., 2019) (Dörgő et al., 2018). One of the main advantages of the proposed methodological development is that it can be applied flexibly in any subject area, such as the representation of the circular economy or industry 4.0 topics in world news. In the table, the column WB_SHORT refers to the ontology number of the World Bank, that empowers the traceability, the column WB_NAME describes the description connected to WB_SHORT.

**Table 12:** Search words applied to proximate SDG 13: Climate action and SDG based on the World Bank Group Topical Taxonomy.

SDG 13: Climate action			
My World 2015			
UNGP_CLIMATE_CHANGE_ACTION (Action taken on climate change)			
WB_SHORT	WB_NAME	WB_SHORT	WB_NAME
WB_587_	Poverty and Climate Change	WB_1844_	Market-Based Climate Change Mitigation
WB_821_	Disaster Risk Reduction	WB_1787_	Natural Habitats
WB_823_	Post Disaster Recovery and Reconstruction	WB_3138_	Natural Disaster
WB_3358_	Man-Made Disasters	WB_1705_	Disaster Preparedness
WB_1770_	Climate Change and Vulnerable Groups	WB_580_	Low Carbon Development
WB_142_	Energy and Water	WB_705_	Economic Shocks and Climate Change
WB_156_	Groundwater Management	WB_1838_	Climate Risk Screening
WB_1798_	Water Pollution	WB_1750_	Climate Change Adaptation Impacts
WB_1998_	Water Economics	WB_1752_	Climate Change Adaptation in Coastal and Marine Areas
WB_1831_	Environmental Crime and Law Enforcement	WB_1753_	Gas-to-Power
WB_849_	Environmental Laws and Regulations	WB_1758_	Transport and Climate Change
WB_158_	Water Resources and Climate Adaptation	WB_1772_	Private Sector and Climate Change
WB_140_	Agricultural Water Management	WB_1773_	Climate Change Impacts
WB_537_	Urban Energy Efficiency	WB_1774_	Climate Forecasting
WB_538_	Energy Efficiency in Industry	WB_1777_	Forests
WB_1756_	Energy and Climate Change	WB_1791_	Air Pollution
WB_520_	PPP in Energy and Power	WB_1795_	Ozone Depleting Substances (ODS)
WB_2673_	Jobs and Climate Change	WB_1837_	Climate Change and Disaster Risk
WB_1075_	Industry Policy	WB_1839_	Ozone Layer Depletion and Climate Change
WB_1979_	Natural Resource Management	WB_1849_	Public Climate Finance
WB_963_	Natural Resources Law	WB_1850_	Private Climate Finance
WB_2639_	Climate Efficient Industries	WB_1878_	Carbon Capture and Storage
WB_582_	Greenhouse Gas Accounting	WB_570_	Early Warning Systems
WB_810_	Climate Change Adaptation in Urban Areas	WB_573_	Climate Risk Management
WB_1841_	Short-lived Climate Pollutants (SLCPs)		

Sustainable development goals are not evenly covered by indicators, and in the case of indicators, there is a significant lack of data, which makes it challenging to monitor their fulfilment. This is especially true of SDG 13, where hardly any measurable indicators are present. This is why it is essential to find additional data sources for which one of the promising tools is the news analysis presented in this research. It is generally true that the SDG indicators do not characterize the awareness and participation of the society, however, the proposed methodological development allows these factors to be taken into account.

### 3.3 Development of the related SQL queries

GDELT uses some of the world's most sophisticated natural-language and data-mining algorithms, including the world's most powerful deep-learning algorithms, to extract and monitor world news. GDELT consists of the Event Database, which captures worldwide activities (events), as well as the Global Knowledge Graph (GKG), which records and entwines people, organizations, locations, themes, taxonomies, sources, tone and events of news into a network. The important attributes of GKG can be seen in Table 13.

**Table 13:** Important attributes of an article for the network creation.

Name	Notation	Description	GDELT attribute
Id	*id*	Identifies an article. This is a unique attribute of all articles	GKGRECORDID
Date	*t*_*i*_	Identifies the publication date of the article	V2.1DATE
Location	*L*_*i*_	List identifying the locations mentioned by the article	V1LOCATIONS
Themes	*D*_*i*_	List identifying the topics	V2ENHANCEDTHEMES
Sentiment	*s*_*i*_	Shows the average tone of the article. This ranges between -100 (extremely negative) and +100 (extremely positive)	V1.5TONE

GKG enables the co-occurrence of people, locations or organizations that empower analysis concerning the relationships between parties to be determined.

GDELT Global Knowledge Graph, which offers scalable, cost-effective, cloud-based opportunities to analyse huge amounts of data, is available as a queryable dataset in the Google BigQuery (GBQ).

GBQ is comprised of a Structured Query Language (SQL)-like syntax, with lots of additional data processing tools, e.g. unnesting (separating columns into multiple rows) and regular expression capturing. This domain-specific language is often used in programming and data management. The queries are based on a schematic SELECT query which captures the main details of the GKG database, namely location, date, topics and tone.

### 3.4 Generation of networks

Network analysis provides a graphical representation of complex patterns of relationships between variables (Hevey, 2018). Therefore, beyond the relationships of countries with regards to sustainability news, the relationships between countries and topics can be described. Furthermore, this method can serve as validation of the selection and categorization of keywords which occurred as a result of expert sampling, as if the keywords are clustering significantly (creating a community), a strong interrelationship can be observed between them and the classification can be confirmed as suitable.

Network analysis carries great potential to:

• explore and visualize relationships;
• identify central nodes - countries/keywords - by defining the number of connections (degrees) to the node or analysing the frequency pathway between other nodes (betweenness);
• detect clustering nodes (communities), e.g. countries systematically involved in a certain topic.

GDELT Global Knowledge Graph allows the co-occurrence exploration of locations (e.g. countries), people as well as themes that appear in news, which can serve as a basis for the network formation.

The analysis is based on the multidimensional network representation of the available information. A multidimensional network can be defined as, nodes (*V*), edges (*E*) and dimensions (*D*):

(1) G=(V,E,D)E={(u,v,d),u,v∈V,d∈D}

An edge is expressed as a connection between two nodes *u* and *v*, in a dimension *d*. In our case, the nodes are countries and the dimensions are sustainable goals as well as their subcategories. The network is generated using the GDELT geolocation, topic recognition and sentiment analysis. The sentiment analysis part will be discussed later, for now, the creation of the simplest network will be concentrated on. GDELT geolocates each article to countries and cities according to the locations mentioned in the article. If an article contains or mentions, two different countries, this is an edge in the network between the two countries. Upon the identification of an edge, the dimension must be accounted for. The network will be multidimensional, therefore, the categories of the article will define which layer the previously mentioned edge appears in.

An article is regarded as a quadruplet of the article's id (*i*), its publication date ($t_{i}$), the identified set of locations mentioned within it ($L_{i}$), the dimensions and tags of the article ($D_{i}$) and its sentiment ($s_{i}$). Table 13 summarizes the connections between the GDELT database and our notations.

(2) $$a_{i}=<i,t_{i},L_{i},D_{i},s_{i}>$$

Based on GDELT, two types of network building are followed. One approach is when the nodes of the network are the topics (integrated SDGs), where the edges are the news, the dimension of the edges is the countries or groups of countries, while the weight of the edges is the number of pieces and/or tone.

The other option is when the nodes of the network are countries and edges are the news of a particular topic, where the weight of the edges can be determined from the number of articles. Networks can be generated directly with developed SQL queries, but can also be generated based on the downloaded database.

The presented tool analyzes the occurrence of sustainable development goals in the news and characterizes the emphasis of the goals based on the assigned World Bank Group Topical Taxonomy categories. A limiting factor of the approach is that the very general categories that are related to a lot of news can mislead the analyst, so it is not enough to interpret aggregated results at the SDG level, but also to control them at the taxonomy level. The tool can be further developed if different taxonomies are not given equal importance to the goals, but the labels that best describe the goals are represented with higher weights.

## 4 Results

The results section shows how world news are related to sustainable development goals. This is the only piece of research that uses a broad-spectrum, systematic, multilingual monitoring tool to describe the sustainability information flow objectively. This methodological development allows to be continuously monitored throughout the world through online queries, thus measuring the social acceptance of SDGs and encouraging participation in terms of their implementation, as well as helping countries around the world to share experiences concerning their problems and successes, which is essential for the implementation of the Agenda. Based on the news, the significance and tone of SDGs in different countries can be modelled on its own. Based on the network analysis, the joint occurrences of the topics can be explored from which the presented approach can be validated. In the news, the spatial allocation of sustainability issues can be measured based on common countries, and countries with similar problems or achievements can be grouped. The analysis can be performed both holistically and in a goal-oriented fashion.

The analysis presented for 2019 can be carried out for any time period. The developed tools and the extracted data are publicly available on the authors' website: abonyilab.com.

In the following, the representation of the proposed method is described through the global overview of the Sustainable Development Goals, after which climate change goal (SDG13) is presented in detail and finally, the SDG interlinkages are illuminated by the interpretation of the generated networks.

### 4.1 The Sustainable Development Goals in the world news

**Figure 2:**
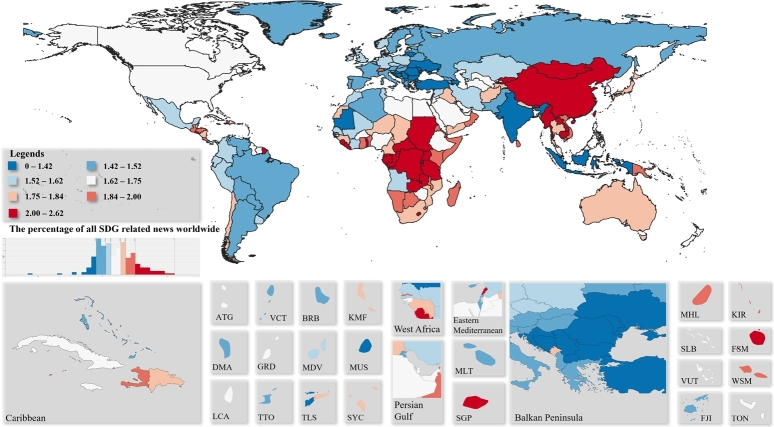
Percentage of SDG-related world's news.

Based on our method it can be determined, that how often the SDGs occur in the news in different countries worldwide. The percentages of world SDG news are summarized in Fig. 2. Cold colours represent lower percentages, while warm colours represent higher percentages. Map cutouts were selected based on the Global Burden of Disease Study (Fullman et al., 2017). For the quantization of the data equal-frequency binning has been applied, in which we divided the dataset into bins that all have an equal number of frequencies.

Fig. 2 shows that the share of sustainability news, even in the most sensitive countries, is only around 2.6%. Sierra Leone leads the flow of information on SDG news with 2.62%, followed by Rwanda with 2.47% and Liberia with 2.44%. There are only 2 countries where the share of SDG-related news is less than 1%, Saint Pierre and Miquelon and Bouvet Island. The distribution shown in Fig. 2 shows that Africa as well as East and Southeast Asia are the general hot spots of SDG news.

In Eastern Europe, the appearance of SDGs in the news lags slightly behind that in Western countries. Considering that the maximum value is around 2.6%, it follows that the scale moves in a relatively narrow range. In Europe, sustainability related news is most common in Switzerland, at 1.94%. For Small Island Developing States (SIDS), the relative frequency varies. There are countries where more SDGs news appear, such as Singapore (2.04%) and Federated States of Micronesia (2.29%) and some where less than Mauritius (1.4%) or Dominica (1.52%).

**Figure 3:**
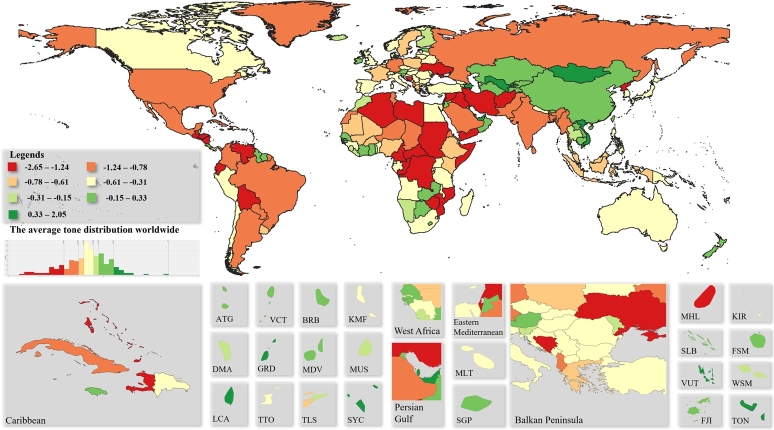
Average tone of SDG related world's news.

The tone of the SDG-related news is illustrated in Fig. 3. For the quantization of the data equal-frequency binning has been applied, in which we divided the dataset into bins that all have an equal number of frequencies. Due to the relatively high number of topic areas, no particular extremes can be observed in Fig. 3. Even the unweighted average tone is represented the aggregate indicator is suitable for presenting the general opinion of the countries concerning the SDGs.

Based on Fig. 3, the most positive (+2.05) is found in Saint Kitts and Nevis, followed by Macau with 1.42. All other positive tones are below +1.00. Other positive countries are Saint Lucia scoring 0.92, Seychelles with 0.82, and then the United Arab Emirates with 0.73. The most pessimistic country is Christmas Island with a tone of -2.65, followed by Libya with -2.44 and Iran with -2.43. A very interesting correlation is observed between Figs. 2 and 3. The most SDG-related news sees the light of day in Africa, but the news is also the most negative here, therefore, a joint analysis of quantity and tone is recommended.

For Small Island Developing States, perceptions of SDG are generally positive (except in Marshall Islands, where -1.25), but overall, the news are more negative in the world as a global picture. One reason for this may be that awareness raising works more effectively if the emphasis is on the negative consequences. In any case, the regional patterns are well understood in the average tone of the news, however, they may be of further interest for analysis of tones at the different SDGs, which is beyond the scope of the present research. Nevertheless, the proposed tool we have developed is directly suitable for conducting such researches. In this case, the average tones can also be interpreted on a wider scale.

The impact of climate change is perhaps the most obvious in the world's news, so the application of the methodology to address this issue will be described separately.

**Figure 4:**
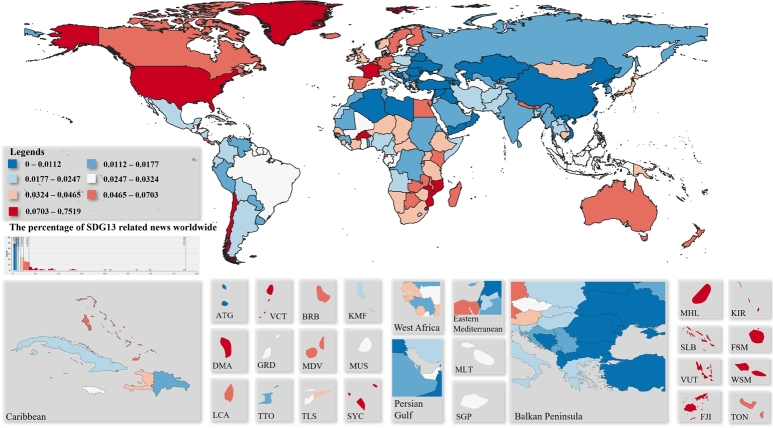
Percentage of climate change action related to world news.

Fig. 4 shows the percentage of world news related to action against climate change in 2019. In the figure, red colours indicate higher frequencies, while colder blue colours indicate lower frequencies. Map cutouts were selected based on the Global Burden of Disease Study (Fullman et al., 2017). For the quantization of the data equal-frequency binning has been applied, in which we divided the dataset into bins that all have an equal number of frequencies.

As a proportion of all the news in the countries of the world, Tuvalu publishes the most news related to climate change, 0.75% to be exact. The second most common is the U.S., where 0.6% of the news is about climate change. The third place is located in the Arctic Ocean, namely the Svalbard archipelago, with 0.54%. The least amount of news concerning climate change appears in Anguilla, 0.003%. In general, the analysis shows that news about climate change is more relevant in small island states. Climate induced risks for small islands includes sea level rise, tropical and extratopical cyclones, changes in rainfall patters and the increase of air and sea surface temperatures (Nurse et al., 2014). This fact has so far not been sufficiently taken into account with regard to the implementation of the 2030 Agenda, as the indicators set for the Climate Action (SDG 13) goal in themselves fail to take into account such groupings of countries.

In general, climate change is more prominent in countries that, due to their geographical location, have a more significant relationship with the seas or oceans. In Europe, France has the most news on climate action, with 0.12%, an order of magnitude higher than the European average.

**Figure 5:**
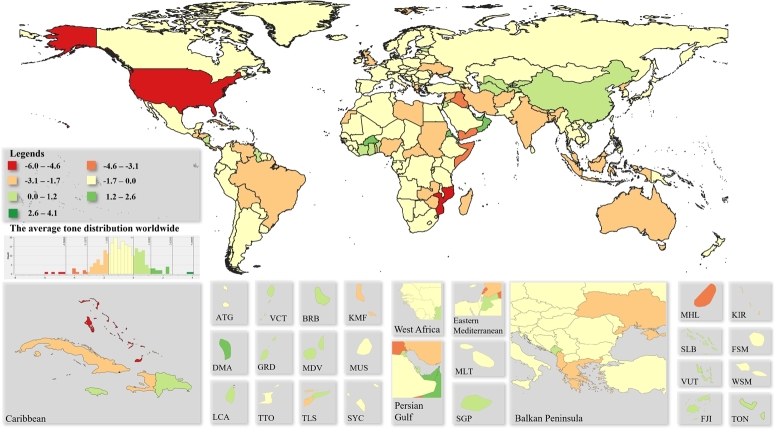
Average tone of climate change action related to world news.

News media play a significant role in informing and engaging citizens in sustainability issues (Östman, 2014), therefore, the tone of news indicates their general opinion. The average tone of the countries can be seen in Fig. 5. Pessimistic countries are denoted in red, while those with a positive attitude are labelled in green and nations that are neutral in yellow. For the quantization of the data equal-frequency binning has been applied, in which we divided the dataset into bins that all have an equal number of frequencies.

According to the news, Cape Verde is the most negative country with regard to climate change, where the average tone is -6. In second place is the United States with a value of -5.5 and in third is The Bahamas with -4.9. The most optimistic country is Aruba, with an average tone of 4.1, followed by Monaco with a value of 3.7 and then Macau with a score of 2.4. It can be seen that the average tone is usually neutral in the world. Further interesting research could be a separate analysis of very negative and very positive news, which can be done directly with the methodology developed in the present research.

### 4.2 The intertwining of the SDGs and world and news

**Figure 6:**
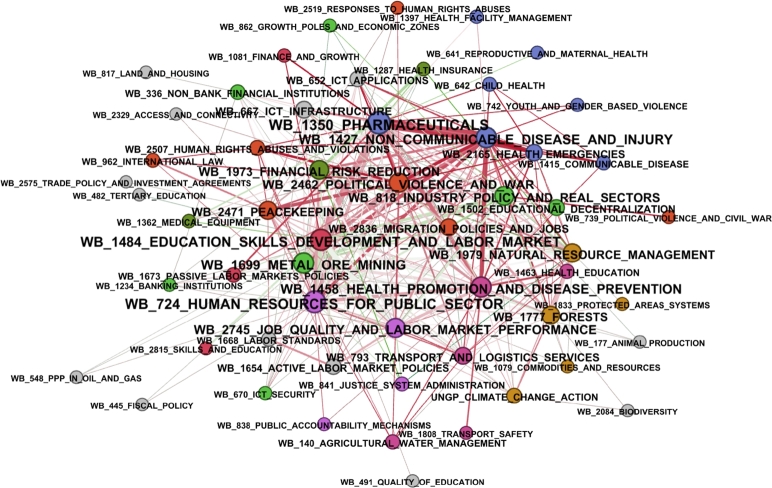
The intertwining of world news and SDGs.

Sustainable development goals are closely interlinked, a fact that is also true for news categories and the typical published news profiles of the countries around the world. In the following, networks are used to illustrate the complex interrelationships of the intertwining of SDGs and news. The network of ontologies characterizing sustainable development goals is illustrated in Fig. 6. In the network, the size of the title of the topics is proportional to its degree of centrality, while the thickness of the links between the issues is proportional to the number of news and the colour shows the average tone.

As is shown in Fig. 6, the identified modules in the network are related to the same topics, which are highlighted by the colours of the nodes, therefore, the connections between the ontologies of the World Bank and the sustainable development goals considered can be validated. The highest degrees of centrality with regard to the nodes in the network are the ‘human resources for public sector’, ‘pharmaceuticals’, ‘metal ore mining’, ‘education skills development and labor market’ and ‘health promotion and disease prevention’. The edges of the network are predominantly red, which means that sustainability news tends to be negative. If the developed queries are used, the development of the network of topic areas over time, the interest in sustainable development, the flow of information, and the speed of responding to environmental problems can all be measured. This research aims to present query-based monitoring as a new option in the field of sustainability assessment. Analyzing changes in the news over time is a promising future research direction.

This research has presented a methodological development based on the combination of news with SDGs that can contribute to the effective delivery of the 2030 Agenda. There are differences between countries around the world in both the proportion and tone of SDG-related news. The proposed methodology is also suitable for holistic and goal-oriented analyses, which were presented across all objectives and on the example of SDG 13. The overall hot spots of sustainability news are mainly in Africa as well as East and Southeast Asia, moreover the most negative tone recorded in Africa. In contrast, in the case of climate change, the United States and small island states are more in focus. These facts outline the foundations for exploring future focal points of SDG 10: Reduced inequalities, nevertheless, they can also help fill in information gaps to identify potential areas for collaboration (SDG 17). Tracking news can also help to better understand the state of technical goals worldwide, such as SDG 6 “Clean water and sanitation,” where SDG indicators focus mainly on infrastructure, while social aspects are highly underrepresented. Analyzing the news provides support for strategists in precisely this objectively difficult area to also measure modern wastewater treatment processes such as heat recovery, enzymatic wastewater treatment or membrane technology which can be measured mainly through news, across the SDG indicators it is not possible.

**Figure 7:**
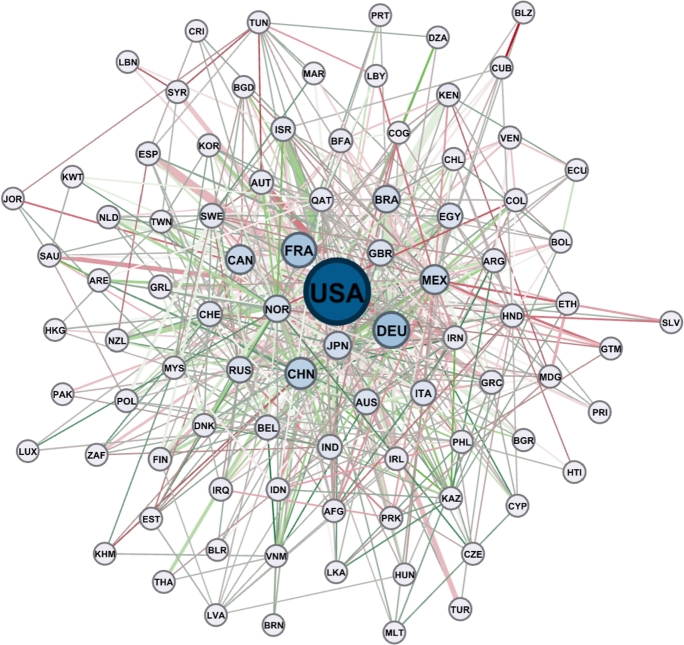
The intertwining of countries worldwide with regard to climate change (SDG 13).

In Fig. 7 the occurrences of countries in the climate action-related (SDG 13) news are represented in a network. The network identifies the intertwining of countries based on countries mentioned together in news with regard to the 13th goal.

The sizes of the nodes are proportional to their occurrence, and the edges represent those mentioned to be in collaboration, where the weight is the number of the news. The colours of the nodes were chosen according to the classes of modularity detected.

Fig. 7 shows that the United States is most often mentioned in the world news about climate change. The U.S. can be grouped with 132 other countries, has a betweenness centrality of 0.525 and is by far the most significant hub in the network. The most weighted edges in the network are France-USA with 3343 news, France-Germany with 3243 news, Canada-USA 3209, UK-US 2368, Russia-USA 2091, Egypt-USA 2052 and China-USA 2015. In terms of nodes, the second most central hub is France, the third is Germany, the fourth is Mexico and the fifth is India.

The provision of information encourages people to participate in environmental protection and, by involving people, makes it possible to maintain the continuity and implementation of sustainability policy in the most cost-effective way. (Iizuka, 2016)

Analyses showed that there was very little difference in the level of environmental quality concerns between developed and developing countries (Iizuka, 2016).

If the environmental preferences of agents are sensitive to environmental pollution and human capital, the economy is able to follow a balanced growth trajectory, with damped oscillations that can be addressed by environmental policy (Constant and Davin, 2019).

The fact that countries play a largely neutral role in the climate change news network is a good indication that we need to make serious efforts to achieve the objectives of the SDGs and the Paris Climate Agreement.

In summary, sustainable development goals can be monitored through news appearing determined by carefully selected keywords. The network of ontologies characterizing sustainable development goals determines their centrality, links and tone. The distribution of the percentage of sustainability news reveals that general hot spots are located in Africa as well as East and Southeast Asia. The average tone of the SDG-related news presenting the general opinion of countries in regard with the issues, for example, considering the average of SDG news, the most positive country is Saint Kitts and Nevis (+2.5), while the most pessimistic is Christmas Island (-2.65). Furthermore, the cooperation of countries in regard with sustainability issues can be determined by the thoughtful analysis of countries co-occurrence in the selected news.

## 5 Conclusion

The major objective of this paper was to determine country profiles as well as interconnections according to the presence of sustainability - assessed through a news-centred network analysis. This method enables the stages of implementation to be determined and serves as a supportive decision-making tool to contribute towards the conscious formation of a sustainable socio-economic ecosystem.

Based on the developed methodology, any SDG can be analyzed in the world news. A comprehensive analysis of 2019 shows that world news are not significantly focus on SDGs, as with all related topics accounting for only roughly 2.5% of the news are covered by SDG-related topics, even in the most sensitive countries. Sustainability news is most prevalent in Africa as well as East and Southeast Asia, with the former having the most negative tone in the region. The proposed methodology will provide essential information for future strategic planning with regard to several goals, in particular in the area of SDG 10, where addressing inequalities is a key challenge.

Through the news, not only holistic but also goal-oriented analyses can be performed. It can be seen that the role of the United States is prominent in SDG 13 and that greater emphasis should be placed on small island states in the future, because the news about climate change is mostly negative in these countries. The analysis shows that more emphasis should be placed in all countries on the appearance of SDGs in the news.

Since countries are also grouped together in terms of different SDG-related ontologies, the relationship between countries can also be analyzed. It has been shown that the United States is one of the most important hubs in the network. The results will contribute to an objective SDG-based analysis of the news, thus helping to track implementation of the 2030 Agenda.

The average tone of SDGs is negative, especially for African countries, while Small Island Developing States have a positive viewpoint on the 2030 Agenda. The most positive countries are Saint Kitts and Nevis +2.05, followed by Macau with 1.42. The most negative news are occurred in Christmas Island with a tone of -2.65, followed by Libya with -2.44 and Iran with -2.43.

We recommend that countries exposed to climate change (especially the SIDS) work together, while tackling the negative appearance of the United States in the news about climate change is an urgent task. The negative perception of African countries about SDGs shows that countries around the world need to put more emphasis on Reduced Inequalities within and among countries (SDG10) goal.

Social acceptance is a key element in the strategic planning of the implementation of 2030 Agenda, so we recommend that national governments inform the public about the tasks, achievements and challenges related to SDGs. A more frequent appearance in the news can be one of the positive drivers for a better understanding of sustainability issues and solutions. The presented monitoring tool can provide useful feedback to decision makers about the awareness of residents and help them learn about their attitudes.

## Declarations

### Author contribution statement

V. Sebestyén: Performed the experiments; Analyzed and interpreted the data; Contributed reagents, materials, analysis tools or data; Wrote the paper.

T. Czvetkó: Analyzed and interpreted the data; Contributed reagents, materials, analysis tools or data; Wrote the paper.

G. Honti: Performed the experiments; Analyzed and interpreted the data; Contributed reagents, materials, analysis tools or data.

J. Abonyi: Conceived and designed the experiments; Performed the experiments; Analyzed and interpreted the data; Contributed reagents, materials, analysis tools or data.

### Funding statement

This work was supported by Széchenyi 2020 (GINOP-2.3.2-15-2016-00016) and NKFIH-872.

### Data availability statement

Data will be made available on request.

### Declaration of interests statement

The authors declare no conflict of interest.

### Additional information

Supplementary content related to this article has been published online at https://doi.org/10.1016/j.heliyon.2021.e06174.

No additional information is available for this paper.

## Appendix Supplementary material

The following Supplementary material is associated with this article:

supplementary.pdf Search words describing Sustainable Development Goals. supplementary.pdf
