# Supplementary material for: The intertwining of world news with Sustainable Development Goals: An effective monitoring tool
Source: Heliyon. 2021 Feb 5;7(2):e06174. doi: 10.1016/j.heliyon.2021.e06174 (PMC7868610; doi:10.1016/j.heliyon.2021.e06174)
Supplement: supplementary.pdf — Search words describing Sustainable Development Goals. [file mmc1.pdf]

Appendix A. Search words used to identify SDG-related articles basis on news analysis

Table A.1: Search words used to identify relevant SDG-related articles based on news analysis

**Each included AND/OR**	
”news”; ”news analysis”; ”media”; ”media analysis”; ”content analysis”; ”sustainable development”; ”SDG”	
**SDGs**	**Specific search words**
Complex	”sustainable development”; ”SDGs”
SDG 1: No poverty	”poverty”; ”vulnerable”
SDG 2: Zero hunger	”hunger”; ”nutrition”; ”underfed”; ”food”; ”food production”; ”agriculture”
SDG 3: Good Health and well-being	”disease”; ”mortality”; ”HIV”; ”Tuberculosis”; ”Malaria”; ”Hepatitis”; ”epidemics”; ”healthcare”; ”addiction”; ”medicine”; ”illness”; ”pollution”; ”hygine”; ”tobacco”; ”well-being”; ”health”
SDG 4: Quality education	”education”; ”childhood development”; ”training”; ”learning environment”
SDG 5: Gender equality	”gender equality”; ”discrimination”; sexual violence”; ”equal opportunities”; ”empowerment of women”
SDG 6: Clean water and sanitation	”clean water and sanitation”; ”access to water”; ”water quality”; ”hazardous chemicals”, ”water pollution”; ”wastewater”; ”water resource management”
SDG 7: Affordable and clean energy	”affordable energy”; ”energy”; ”renewable energy”; ”energy efficiency”; ”clean energy”; ”sustainable energy”
SDG 8: Decent work and economic growth	”economic growth”; ”productivity”; ”job”; ”decent work”; ”entreprenuership”; ”innovation”; ”SMEs”; ”resource efficiency”; ”employment”; ”unemployment”; ”labour market”; ”sustainable tourism”
SDG 9: Industry, innovation and ifrastructure	”industry”; ”innovation”; ”sustainable infrastructure”; ”manufacturing”; ”research and innovation”; ”information and communication infrastructure”; ”ICT”
SDG 10: Reduced inequalities	”reduced inequalities”; ”inequality”; ”equal opportunity”; ”discrimination”; ”equality”; ”migration”;
SDG 11: Sustainable cities and communities	”sustainable cities”; ”affordable housing”; ”urban living”; ”sustainable transport”; ”heritage”; ”urban and regional development”
SDG 12: Responsible consumption and production	”sustainable consumption”; ”natural resources”; ”resource efficiency”; ”material consumption”; ”domestic material”; ”hazardous waste”; ”recycling”; ”fossil fuel”
SDG 13: Climate action	”climate action”; ”climate change”; ”natural disaster”; ”greenhouse gas”; ”CO2 emission”; ”global warming”
SDG 14: Life below water	”marine pollution”; ”nutrient pollution”; ”coastal ecosystem”; ”oceans”; ”acidification”; ”overfishing”; ”fishing”; ”biodiversity”
SDG 15: Life on land	”forest”; ”freshwater”; ”biodiversity”; ”desertification”; ”mountain”; ”wildlife”; ”on land”
SDG 16: Peace, justice and strong institutions	”peace”; ”justice”; ”strong institutions”; ”human rights”; ”violence”; ”death”; ”victims”; ”safety”; ”conflicts”; ”protest”; ”governance”; ”public access to information”; ”kidnapping”; ”murder”
SDG 17: Partnership fot he goals	”partnership”; ”agreement”; ”foreign direct investment”; ”South-South”; ”North-South”; ”cooperation”; ”global partnership”; ”capacity-building”

Appendix B. Defined search words describing SDG goals

Table B.2: Search words applied to proximate SDG 1: No poverty and SDG 2: Zero hunger, based on the World Bank Group Topical Taxonomy and the My World 2015 survey

**SDG 1: No poverty**	
**WB_SHORT**	**WB_NAME**
WB_1091_	Growth, Poverty and Inequality
WB_1151_	Multi-dimensional Poverty
WB_1152_	Economic and Social Mobility
WB_1946_	Real-time Poverty Data
WB_587_	Poverty and Climate Change
WB_709_	Poverty and Social Impact Analysis
WB_712_	Transport and Poverty
WB_491_	Quality of Education
WB_711_	Fiscal Incidence Analysis
WB_2788_	Labor Market Institutions and Policies
WB_1166_	Spatial Inequality
WB_2668_	Income Inequality
WB_1167_	Equality of Opportunities
WB_1093_	Social Protection and Growth
WB_462_	Growth and Lagging Regions
WB_821_	Disaster Risk Reduction
WB_823_	Post Disaster Recovery and Reconstruction
WB_3358_	Man-Made Disasters
WB_756_	Vulnerable Groups
WB_1770_	Climate Change and Vulnerable Groups
WB_1162_	Poverty, Nutrition and Food Security
WB_1165_	Poverty and Fiscal, Financial and Economic Crisis
WB_807_	Slum Upgrading

**SDG 2: Zero hunger**	
**My World 2015**	
UNGP_AFFORDABLE_NUTRITIOUS_FOOD (Affordable and nutritious food)	
**WB_SHORT**	**WB_NAME**
WB_1162_	Poverty, Nutrition and Food Security
WB_646_	Food Fortification
WB_703_	Economic Shocks and Food Security
WB_1960_	Farm Inputs
WB_177_	Animal Production
WB_1971_	On-Farm Storage
WB_1059_	Agricultural Finance
WB_171_	Agricultural Laws and Regulations
WB_1950_	Agriculture Technology
WB_1963_	Responsible Agricultural Investment
WB_1978_	Agriculture and Related Subsidies
WB_433_	Gender and Agriculture

Table B.3: Search words applied to proximate SDG 3: Good health and well-being and SDG 4: Quality education, based on the World Bank Group Topical Taxonomy and the My World 2015 survey

**SDG 3: Good health and well-being**	
**My World 2015**	
UNGP_JOB_OPPORTUNITIES_WORKING_CONDITIONS (Better healthcare)	
**WB_SHORT**	**WB_NAME**
WB_1162_	Poverty, Nutrition and Food Security
WB_1287_	Health Insurance
WB_1288_	Health Equity and Access
WB_1304_	Health Provider Payment
WB_1320_	Quality of Health Care Delivery
WB_1397_	Health Facility Management
WB_1458_	Health Promotion and Disease Prevention
WB_1792_	Environmental Health
WB_2165_	Health Emergencies
WB_631_	Human Resources for Health
WB_641_	Reproductive and Maternal Health
WB_642_	Child Health
WB_684_	Health Management Information Systems
WB_1414_	Burden of Disease
WB_1415_	Communicable Disease
WB_1427_	Non-Communicable Disease and Injury
WB_1316_	Urban Health
WB_1317_	Rural Health
WB_1319_	Health Facilities Restructuring
WB_1327_	Community Based Interventions in Health
WB_1328_	Basic Package of Health Services
WB_1396_	Health Provider Networks
WB_1463_	Health Education
WB_2155_	Financial Sustainability for Health
WB_2159_	Essential Package of Health Services
WB_2160_	Procurement of Health Technologies
WB_2181_	Health Basket Finance
WB_2681_	Jobs and Health Epidemics
WB_626_	Health Financing through General Taxation
WB_628_	Health Care Resource Allocation
WB_920_	Gender and Health
WB_1456_	Maternal Mortality and Morbidity
WB_1289_	Fiscal Sustainability for Health
WB_1290_	Fiscal Space for Health
WB_1296_	Aid Flows for Health
WB_1308_	Palliative Care
WB_1350_	Pharmaceuticals
WB_1362_	Medical Equipment

**SDG 4: Quality education**	
**My World 2015**	
UNGP_EDUCATION (A good education)	
**WB_SHORT**	**WB_NAME**
WB_491_	Quality of Education
WB_1167_	Equality of Opportunities
WB_1463_	Health Education
WB_1468_	Social Demand for Education
WB_1484_	Education, Skills Development and Labor Market
WB_1499_	Parent, Family, Community Involvement in Education
WB_1502_	Educational Decentralization
WB_1504_	Education Management Information Systems
WB_1513_	Education Policy and Planning
WB_1559_	Adult Basic Education
WB_2124_	Economic Analysis of Education
WB_2127_	Education Access and Equity
WB_2815_	Skills and Education
WB_480_	Primary Education
WB_481_	Secondary Education
WB_482_	Tertiary Education
WB_483_	Vocational Education and Training
WB_494_	Education and ICT
WB_498_	Education Finance
WB_1510_	School Administration
WB_1523_	Teachers Management

Table B.4: Search words applied to proximate SDG 5: Gender equality, and SDG 6: Clean water and sanitation, based on the World Bank Group Topical Taxonomy and the My World 2015 survey

**SDG 5: Gender equality**	
**MYWorld 2015**	
UNGP_GENDER_EQUALITY (Equality between men and women)	
**WB_SHORT**	**WB_NAME**
WB_1167_	Equality of Opportunities
WB_1843_	Social inclusion of women
WB_2668_	Income Inequality
WB_2899_	Women and Corporate Leadership
WB_3025_	Gender and ICT
WB_3026_	Gender and Water Supply and Sanitation
WB_3029_	Gender and Disaster Risk Management
WB_3105_	Gender and Growth
WB_433_	Gender and Agriculture
WB_616_	Women and Access to Justice
WB_618_	Gender and Employment
WB_619_	Gender and Energy
WB_620_	Gender and Transport
WB_742_	Youth and Gender-Based Violence
WB_914_	Gender and Finance
WB_915_	Gender and Land
WB_916_	Female-Headed Households
WB_918_	Gender and Mining
WB_920_	Gender and Health
WB_925_	Inequality under Law
WB_934_	Gender and Youth

**SDG 6: Clean water and sanitation**	
**MYWorld 2015**	
UNGP_CLEAN_WATER_SANITATION (Access to clean water and sanitation)	
**WB_SHORT**	**WB_NAME**
WB_1000_	Water Management Structures
WB_1063_	Water Allocation and Water Supply
WB_1064_	Water Demand Management
WB_1220_	Surface Water Management
WB_138_	Water Supply
WB_140_	Agricultural Water Management
WB_142_	Energy and Water
WB_155_	Watershed Management
WB_156_	Groundwater Management
WB_157_	Environmental Water Use and Catchment Protection
WB_158_	Water Resources and Climate Adaptation
WB_159_	Transboundary Water
WB_1798_	Water Pollution
WB_1805_	Waterways
WB_1831_	Environmental Crime and Law Enforcement
WB_1998_	Water Economics
WB_2006_	PPP in Water Supply and Sanitation
WB_2007_	Water Safety Plans
WB_2008_	Water Treatment
WB_2009_	Water Quality Monitoring
WB_2199_	Sanitation Engineering
WB_3026_	Gender and Water Supply and Sanitation
WB_849_	Environmental Laws and Regulations

Table B.5: Search words applied to proximate SDG 7: Affordable and clean energy, and SDG 8: Decent work and economic growth, based on the World Bank Group Topical Taxonomy and the My World 2015 survey

**SDG 7: Affordable and clean energy**	
**My World 2015**	
UNGP_RELIABLE_ENERGY (Reliable energy)	
**WB_SHORT**	**WB_NAME**
WB_142_	Energy and Water
WB_1831_	Environmental Crime and Law Enforcement
WB_849_	Environmental Laws and Regulations
WB_537_	Urban Energy Efficiency
WB_538_	Energy Efficiency in Industry
WB_2273_	Upstream Oil and Gas
WB_540_	Governance for Oil and Gas
WB_541_	Government Institutions for Oil and Gas
WB_544_	Mid and Downstream Oil and Gas
WB_548_	PPP in Oil and Gas
WB_1699_	Metal Ore Mining
WB_555_	Artisanal and Small-Scale Mining
WB_556_	Mining Fiscal Policies and Revenue Collection
WB_557_	Governance for Mining
WB_558_	Government Institutions for Mining
WB_2279_	Energy Resource Mobilization
WB_2280_	Energy Risk Mitigation
WB_509_	Nuclear Energy
WB_528_	Solar Energy
WB_529_	Wind Energy
WB_530_	Geothermal Energy
WB_531_	Biomass Energy
WB_532_	Biofuels Energy
WB_534_	Efficient Energy Supply
WB_536_	Energy Efficient Transport
WB_1697_	Energy Efficiency Finance
WB_1703_	Power Distribution
WB_1704_	Power Systems Planning
WB_1756_	Energy and Climate Change
WB_2619_	Results-Based Financing for Energy
WB_2620_	Household Energy Expenditures
WB_2621_	Household Access to Heating Fuels
WB_520_	PPP in Energy and Power
WB_522_	Grid Connected Household Electrification
WB_523_	Household Access to Modern Cooking Fuels
WB_524_	Off Grid and Mini-Grid Household Electrification
WB_510_	Power Transmission
WB_511_	Coal-Fired Power
WB_512_	Gas-Fired Power
WB_513_	Oil-Fired Power
WB_516_	Power Sector Economics
WB_517_	Electricity Market Design and Regulation

**SDG 8: Decent work and economic growth**	
**My World 2015**	
UNGP_JOB_OPPORTUNITIES_EMPLOYMENT (Better job opportunities)	
UNGP_SUPPORT_FOR_PEOPLE_WHO_CANT_WORK (Support for people who can't work)	
**WB_SHORT**	**WB_NAME**
WB_1091_	Growth, Poverty and Inequality
WB_1152_	Economic and Social Mobility
WB_2788_	Labor Market Institutions and Policies
WB_1093_	Social Protection and Growth
WB_1165_	Poverty and Fiscal, Financial and Economic Crisis
WB_1059_	Agricultural Finance
WB_1484_	Education, Skills Development and Labor Market
WB_1513_	Education Policy and Planning
WB_2124_	Economic Analysis of Education
WB_498_	Education Finance
WB_1998_	Water Economics
WB_1088_	Macroeconomic Stability and Growth
WB_1096_	Macroeconomic Sustainability
WB_2111_	Competition Economics
WB_3119_	Structural Macroeconometric Models
WB_331_	Economic Transparency
WB_836_	Political Economy of Reform
WB_862_	Growth Poles and Economic Zones
WB_1081_	Finance and Growth
WB_2864_	Firm Dynamics and Productivity Growth
WB_377_	Firm Innovation, Productivity and Growth
WB_473_	Growth Diagnostics
WB_475_	Jobs and Growth
WB_1654_	Active Labor Market Policies
WB_1668_	Labor Standards
WB_1673_	Passive Labor Market Policies
WB_2745_	Job Quality and Labor Market Performance
WB_1249_	Access to Finance
WB_2262_	PPP Finance
WB_370_	Trade Finance
WB_457_	Subnational Debt and Finance
WB_716_	Managing Public Finances
WB_882_	MSME Finance
WB_2673_	Jobs and Climate Change
WB_2683_	Changing Nature of Jobs
WB_2773_	Fiscal Policy and Jobs
WB_2826_	Trade Policies and Jobs
WB_2832_	Investment Policies and Jobs
WB_2836_	Migration Policies and Jobs
WB_2875_	Urbanization and Jobs
WB_848_	Labor and Employment Laws and Regulations
WB_2886_	Aging and Jobs
WB_2889_	Minorities and Disenfranchised Groups and Jobs
WB_701_	Jobs and Poverty
WB_1192_	Impact of Trade
WB_2563_	Trade and FDI Promotion Infrastructure
WB_2575_	Trade Policy and Investment Agreements
WB_2601_	Trade Linkages, Spillovers and Connectivity
WB_865_	Trade Corridors
WB_1747_	Product Market Regulation and Competition Advocacy
WB_1075_	Industry Policy
WB_1678_	Policy Planning, Design and Evaluation
WB_2770_	Monetary Policy and Employment
WB_279_	ICT Strategy, Policy and Regulation
WB_288_	Telecommunications Sector Policy and Regulation
WB_378_	Innovation and Technology Policy
WB_445_	Fiscal Policy
WB_769_	Investment Policy and Promotion
WB_818_	Industry Policy and Real Sectors
WB_1095_	Political and Institutional Sustainability
WB_1095_	Political and Institutional Sustainability
WB_1071_	Economic Growth Project Analysis
WB_1076_	Monetary Policy and Growth
WB_1079_	Commodities and Resources
WB_1080_	Competition Policy and Growth
WB_1082_	Human Capital and Growth
WB_1083_	Infrastructure and Growth
WB_1084_	Innovation, Technology Transfer and Diffusion
WB_1089_	Structural Transformation for Growth
WB_1106_	Macroeconomic Performance
WB_1132_	Labor Market Regulation

Table B.6: Search words applied to proximate SDG 9: Industry, innovation and infrastructure, and SDG 10: Reduced inequalities, based on the World Bank Group Topical Taxonomy and the My World 2015 survey

**SDG 9: Industry, innovation and infrastructure**	
**My World 2015**	
UNGP_TRANSPORTATION_ROADS (Better transport and roads)	
UNGP_PHONE_INTERNET_ACCESS_SLOW (Phone and internet access speed)	
UNGP_PHONE_INTERNET_ACCESS_COST (Phone and internet access cost)	
**WB_SHORT**	**WB_NAME**
WB_494_	Education and ICT
WB_2864_	Firm Dynamics and Productivity Growth
WB_377_	Firm Innovation, Productivity and Growth
WB_475_	Jobs and Growth
WB_2563_	Trade and FDI Promotion Infrastructure
WB_1075_	Industry Policy
WB_279_	ICT Strategy, Policy and Regulation
WB_288_	Telecommunications Sector Policy and Regulation
WB_378_	Innovation and Technology Policy
WB_818_	Industry Policy and Real Sectors
WB_1083_	Infrastructure and Growth
WB_1945_	Knowledge-based Capital for Innovation
WB_2401_	ICT Innovation Methodologies
WB_689_	Science, Technology and Innovation
WB_351_	Payment and Market Infrastructure
WB_667_	ICT Infrastructure
WB_801_	PPP in Infrastructure and Social Sectors
WB_2375_	ICT Methods and Procedures
WB_652_	ICT Applications
WB_670_	ICT Security
WB_2651_	SME Development
WB_2329_	Access and Connectivity
WB_2607_	Competitiveness Diagnostics
WB_614_	Competitive Cities
WB_1276_	Institutions and Growth
WB_163_	Low-Emissions Transport
WB_281_	ICT Industry and Services
WB_970_	Industry Laws and Regulations
WB_1811_	Transport Infrastructure and Environment
WB_2961_	Resilient Infrastructure
WB_514_	Regional and Cross-Border Infrastructure

**SDG 10: Reduced inequalities**	
**My World 2015**	
UNGP_FREEDOM_FROM_DISCRIMINATION (Freedom from discrimination)	
**WB_SHORT**	**WB_NAME**
WB_1091_	Growth, Poverty and Inequality
WB_1152_	Economic and Social Mobility
WB_1166_	Spatial Inequality
WB_2668_	Income Inequality
WB_1167_	Equality of Opportunities
WB_1093_	Social Protection and Growth
WB_1165_	Poverty and Fiscal, Financial and Economic Crisis
WB_2124_	Economic Analysis of Education
WB_1843_	Social inclusion of women
WB_2899_	Women and Corporate Leadership
WB_616_	Women and Access to Justice
WB_925_	Inequality under Law
WB_2620_	Household Energy Expenditures
WB_1088_	Macroeconomic Stability and Growth
WB_1096_	Macroeconomic Sustainability
WB_2111_	Competition Economics
WB_331_	Economic Transparency
WB_862_	Growth Poles and Economic Zones
WB_377_	Firm Innovation, Productivity and Growth
WB_473_	Growth Diagnostics
WB_475_	Jobs and Growth
WB_2836_	Migration Policies and Jobs
WB_701_	Jobs and Poverty
WB_2204_	In-Migration
WB_1109_	Expenditure Analysis and Tools
WB_1245_	Financial Vulnerability and Risks
WB_1255_	Financial Capability
WB_1973_	Financial Risk Reduction
WB_2077_	Financial Supervision for Integrity
WB_2078_	Financial Inclusion and Financial Integrity
WB_355_	International Remittances
WB_374_	Financial Sector and Social Inclusion
WB_853_	Financial Laws and Regulations

Table B.7: Search words applied to proximate SDG 11: Sustainable cities and communities, and SDG 12: Responsible consumption and production, based on the World Bank Group Topical Taxonomy and the My World 2015 survey

**SDG 11: Sustainable cities and communities**	
**WB_SHORT**	**WB_NAME**
WB_821_	Disaster Risk Reduction
WB_807_	Slum Upgrading
WB_631_	Human Resources for Health
WB_1316_	Urban Health
WB_1798_	Water Pollution
WB_537_	Urban Energy Efficiency
WB_538_	Energy Efficiency in Industry
WB_1096_	Macroeconomic Sustainability
WB_2875_	Urbanization and Jobs
WB_279_	ICT Strategy, Policy and Regulation
WB_1095_	Political and Institutional Sustainability
WB_1079_	Commodities and Resources
WB_2401_	ICT Innovation Methodologies
WB_667_	ICT Infrastructure
WB_652_	ICT Applications
WB_670_	ICT Security
WB_281_	ICT Industry and Services
WB_867_	Cities and Conflict
WB_872_	Smart Cities
WB_1808_	Transport Safety
WB_1826_	Transport Policy
WB_791_	Transport Impact on the Environment
WB_793_	Transport and Logistics Services
WB_804_	Urban Pollution
WB_1789_	Physical and Cultural Resources
WB_1979_	Natural Resource Management
WB_724_	Human Resources for Public Sector
WB_963_	Natural Resources Law
WB_2639_	Climate Efficient Industries
WB_837_	Open Government and Transparency
WB_822_	Disaster Risk Assessment
WB_408_	Green Buildings
WB_582_	Greenhouse Gas Accounting
WB_1720_	City Strategies and Territorial Planning
WB_808_	Urban Participatory Planning
WB_810_	Climate Change Adaptation in Urban Areas
WB_811_	Disaster Resilient Cities
WB_814_	City Systems
WB_817_	Land and Housing
WB_1727_	Transport and Urban Development
WB_1781_	Urban Ecosystems
WB_1836_	Urbanization Reviews
WB_1797_	Solid Waste
WB_1828_	Municipal Waste Management

**SDG 12: Responsible consumption and production**	
**WB_SHORT**	**WB_NAME**
WB_177_	Animal Production
WB_641_	Reproductive and Maternal Health
WB_1484_	Education, Skills Development and Labor Market
WB_142_	Energy and Water
WB_156_	Groundwater Management
WB_1798_	Water Pollution
WB_1998_	Water Economics
WB_1831_	Environmental Crime and Law Enforcement
WB_849_	Environmental Laws and Regulations
WB_1000_	Water Management Structures
WB_1064_	Water Demand Management
WB_140_	Agricultural Water Management
WB_537_	Urban Energy Efficiency
WB_538_	Energy Efficiency in Industry
WB_2864_	Firm Dynamics and Productivity Growth
WB_377_	Firm Innovation, Productivity and Growth
WB_1747_	Product Market Regulation and Competition Advocacy
WB_279_	ICT Strategy, Policy and Regulation
WB_2375_	ICT Methods and Procedures
WB_652_	ICT Applications
WB_281_	ICT Industry and Services
WB_970_	Industry Laws and Regulations
WB_2639_	Climate Efficient Industries
WB_1797_	Solid Waste
WB_1828_	Municipal Waste Management
WB_174_	Crop Production
WB_1017_	Consumer Protection Law
WB_364_	Consumer Protection
WB_1793_	Hazardous Wastes
WB_1133_	Productivity Enhancing Policy
WB_2615_	Diversification of Production and Exports
WB_2249_	PPP Accounting and Reporting

Table B.8: Search words applied to proximate SDG 13: Climate action, and SDG 14: Life below water based on the World Bank Group Topical Taxonomy and the My World 2015 survey

**SDG 13: Climate action**	
**My World 2015**	
UNGP_CLIMATE_CHANGE_ACTION (Action taken on climate change)	
**WB_SHORT**	**WB_NAME**
WB_587_	Poverty and Climate Change
WB_821_	Disaster Risk Reduction
WB_823_	Post Disaster Recovery and Reconstruction
WB_3358_	Man-Made Disasters
WB_1770_	Climate Change and Vulnerable Groups
WB_142_	Energy and Water
WB_156_	Groundwater Management
WB_1798_	Water Pollution
WB_1998_	Water Economics
WB_1831_	Environmental Crime and Law Enforcement
WB_849_	Environmental Laws and Regulations
WB_158_	Water Resources and Climate Adaptation
WB_140_	Agricultural Water Management
WB_537_	Urban Energy Efficiency
WB_538_	Energy Efficiency in Industry
WB_1756_	Energy and Climate Change
WB_520_	PPP in Energy and Power
WB_2673_	Jobs and Climate Change
WB_1075_	Industry Policy
WB_1979_	Natural Resource Management
WB_963_	Natural Resources Law
WB_2639_	Climate Efficient Industries
WB_582_	Greenhouse Gas Accounting
WB_810_	Climate Change Adaptation in Urban Areas
WB_1841_	Short-lived Climate Pollutants (SLCPs)
WB_1844_	Market-Based Climate Change Mitigation
WB_1787_	Natural Habitats
WB_3138_	Natural Disaster
WB_1705_	Disaster Preparedness
WB_580_	Low Carbon Development
WB_705_	Economic Shocks and Climate Change
WB_1838_	Climate Risk Screening
WB_1750_	Climate Change Adaptation Impacts
WB_1752_	Climate Change Adaptation in Coastal and Marine Areas
WB_1753_	Gas-to-Power
WB_1758_	Transport and Climate Change
WB_1772_	Private Sector and Climate Change
WB_1773_	Climate Change Impacts
WB_1774_	Climate Forecasting
WB_1777_	Forests
WB_1791_	Air Pollution
WB_1795_	Ozone Depleting Substances (ODS)
WB_1837_	Climate Change and Disaster Risk
WB_1839_	Ozone Layer Depletion and Climate Change
WB_1849_	Public Climate Finance
WB_1850_	Private Climate Finance
WB_1878_	Carbon Capture and Storage
WB_570_	Early Warning Systems
WB_573_	Climate Risk Management

**SDG 14: Life below water**	
**My World 2015**	
UNGP_FORESTS_RIVERS_OCEANS (Protecting forests, rivers, and oceans)	
**WB_SHORT**	**WB_NAME**
WB_821_	Disaster Risk Reduction
WB_823_	Post Disaster Recovery and Reconstruction
WB_3358_	Man-Made Disasters
WB_142_	Energy and Water
WB_156_	Groundwater Management
WB_159_	Transboundary Water
WB_1798_	Water Pollution
WB_1998_	Water Economics
WB_1831_	Environmental Crime and Law Enforcement
WB_849_	Environmental Laws and Regulations
WB_1000_	Water Management Structures
WB_138_	Water Supply
WB_140_	Agricultural Water Management
WB_2673_	Jobs and Climate Change
WB_1075_	Industry Policy
WB_818_	Industry Policy and Real Sectors
WB_2639_	Climate Efficient Industries
WB_1844_	Market-Based Climate Change Mitigation
WB_3138_	Natural Disaster
WB_1705_	Disaster Preparedness
WB_1752_	Climate Change Adaptation in Coastal and Marine Areas
WB_1799_	Marine Transport
WB_596_	Coastal and Marine Ecosystems
WB_1833_	Protected Areas Systems
WB_605_	Wildlife Resources

Table B.9: Search words applied to proximate SDG 15: Life on land and SDG 16: Peace, justice and strong institutions, based on the World Bank Group Topical Taxonomy and the My World 2015 survey

**SDG 15: Life on land**	
**My World 2015**	
UNGP_FORESTS_RIVERS_OCEANS (Protecting forests, rivers, and oceans)	
**WB_SHORT**	**WB_NAME**
WB_821_	Disaster Risk Reduction
WB_823_	Post Disaster Recovery and Reconstruction
WB_3358_	Man-Made Disasters
WB_1059_	Agricultural Finance
WB_1950_	Agriculture Technology
WB_1831_	Environmental Crime and Law Enforcement
WB_849_	Environmental Laws and Regulations
WB_140_	Agricultural Water Management
WB_1979_	Natural Resource Management
WB_963_	Natural Resources Law
WB_1781_	Urban Ecosystems
WB_3138_	Natural Disaster
WB_1705_	Disaster Preparedness
WB_1777_	Forests
WB_1833_	Protected Areas Systems
WB_605_	Wildlife Resources
WB_888_	Land Tenure
WB_593_	Mountain Ecosystems
WB_2084_	Biodiversity
WB_1779_	Modified Ecosystems
WB_1830_	Ecosystem Policies and Institutions
WB_576_	Ecosystem-Based Adaptation (EBA)
WB_1057_	Sustainable Forest Management
WB_1832_	Mainstreaming and Production Landscape

**SDG 16: Peace, justice and strong institutions**	
**My World 2015**	
UNGP_POLITICAL_FREEDOMS (Political freedoms)	
UNGP_CRIME_VIOLENCE (Protection against crime and violence)	
UNGP_HEALTHCARE (An honest and responsive government)	
**WB_SHORT**	**WB_NAME**
WB_2788_	Labor Market Institutions and Policies
WB_742_	Youth and Gender-Based Violence
WB_616_	Women and Access to Justice
WB_1096_	Macroeconomic Sustainability
WB_331_	Economic Transparency
WB_716_	Managing Public Finances
WB_1095_	Political and Institutional Sustainability
WB_1276_	Institutions and Growth
WB_867_	Cities and Conflict
WB_837_	Open Government and Transparency
WB_1720_	City Strategies and Territorial Planning
WB_2462_	Political Violence and War
WB_2507_	Human Rights Abuses and Violations
WB_1234_	Banking Institutions
WB_2115_	Institutional Building for Competition
WB_336_	Non-Bank Financial Institutions
WB_1385_	Governance and Stewardship
WB_417_	Corporate Governance
WB_1650_	Public Employment Services
WB_718_	Public Investment Management
WB_838_	Public Accountability Mechanisms
WB_720_	Tax and Revenue Policy and Administration
WB_2514_	Human Rights Norms and Mechanisms
WB_2519_	Responses to Human Rights Abuses
WB_2471_	Peacekeeping
WB_1014_	Criminal Justice
WB_841_	Justice System Administration
WB_1937_	Civil Justice
WB_1938_	Informal Justice
WB_1939_	Public Trust and Confidence in the Justice System
WB_941_	Traditional Justice and Customary Law
WB_942_	Access to Justice
WB_739_	Political Violence and Civil War
WB_930_	Violence Prevention
WB_931_	Societal Dimensions of Violence and Conflict
WB_733_	Social Accountability
WB_1783_	Environmental Governance
WB_834_	Governance Indicators

Table B.10: Search words applied to proximate SDG 17: Partnership for the goals, based on the World Bank Group Topical Taxonomy and the My World 2015 survey

**SDG 17: Partnership for the goals**	
**WB_SHORT**	**WB_NAME**
WB_1059_	Agricultural Finance
WB_1513_	Education Policy and Planning
WB_498_	Education Finance
WB_1088_	Macroeconomic Stability and Growth
WB_1096_	Macroeconomic Sustainability
WB_1081_	Finance and Growth
WB_2262_	PPP Finance
WB_370_	Trade Finance
WB_457_	Subnational Debt and Finance
WB_716_	Managing Public Finances
WB_2832_	Investment Policies and Jobs
WB_2575_	Trade Policy and Investment Agreements
WB_1075_	Industry Policy
WB_1678_	Policy Planning, Design and Evaluation
WB_279_	ICT Strategy, Policy and Regulation
WB_378_	Innovation and Technology Policy
WB_769_	Investment Policy and Promotion
WB_818_	Industry Policy and Real Sectors
WB_1106_	Macroeconomic Performance
WB_355_	International Remittances
WB_1826_	Transport Policy
WB_837_	Open Government and Transparency
WB_718_	Public Investment Management
WB_720_	Tax and Revenue Policy and Administration
WB_3136_	South-South Cooperation
WB_1977_	Information and Statistics (AMIS)
WB_1815_	Fuel Taxes
WB_1261_	International Standards on Combating Money Laundering and the Financing of Terrorism and Proliferation
WB_962_	International Law
WB_3134_	Sustainable Development Goals

Appendix C. Abbreviations

ATG	Antigua and Barbuda
BRB	Barbados
D	dimension
DMA	Dominica
E	edges
FJI	Fiji
FSM	Micronesia (Federated States of)
G	multidimensional network
GBQ	Google Big Query
GDELT	Global Database of Events, Language and Tone
GIS	Geographic Information System
GKG	Global Knowledge Graph
GRD	Grenada
i	Article ID
KIR	Kiribati
KMF	Comoro Islands
LCA	St. Lucia
Li	Location
MDV	Maldives
MHL	Marshall Islands
MLT	Malta
MUS	Mauritius
SDG	Sustainable Development Goal
SGP	Singapore
si	Sentiment
SLB	Solomon Islands
SQL	Structured Query Language
SYC	Seychelles
ti	Publication date
TLS	Timor-Leste
TON	Tonga
TTO	Trinidad and Tobago
V	nodes
VCT	St. Vincent and the Grenadines
VNR	Voluntary National Review
VUT	Vanuatu
WBG	World Bank Group
WSM	Samoa
